# Diversity and functional structure of soil animal communities suggest soil animal food webs to be buffered against changes in forest land use

**DOI:** 10.1007/s00442-021-04910-1

**Published:** 2021-04-14

**Authors:** Melanie M. Pollierer, Bernhard Klarner, David Ott, Christoph Digel, Roswitha B. Ehnes, Bernhard Eitzinger, Georgia Erdmann, Ulrich Brose, Mark Maraun, Stefan Scheu

**Affiliations:** 1 J.F. Blumenbach Institute of Zoology and Anthropology, University of Göttingen, Untere Karspüle 2, 37073 Göttingen, Germany; 2 Centre for Biodiversity Monitoring (Zbm), Zoological Research Museum Alexander Koenig, Adenauerallee 160, 53113 Bonn, Germany; 3 German Centre for Integrative Biodiversity Research (iDiv) Halle-Jena-Leipzig, Deutscher Platz 5e, 04103 Leipzig, Germany; 4 Institute of Biodiversity, Friedrich-Schiller-University Jena, Dornburger Str. 159, 07743 Jena, Germany; 5 Centre of Biodiversity and Sustainable Land Use, University of Göttingen, Büsgenweg 1, 37077 Göttingen, Germany

**Keywords:** Forest management, Disturbance, Structural equation modelling, Soil pH, Invertebrates

## Abstract

Forest soil and litter is inhabited by a diverse community of animals, which directly and indirectly rely on dead organic matter as habitat and food resource. However, community composition may be driven by biotic or abiotic forces, and these vary with changes in habitat structure and resource supply associated with forest land use. To evaluate these changes, we compiled comprehensive data on the species composition of soil animal communities and environmental factors in forest types varying in land-use intensity in each of three regions in Germany, i.e., coniferous, young managed, old managed, and unmanaged beech forests. Coniferous forests featured high amounts of leaf litter and low microbial biomass concentrations contrasting in particular unmanaged beech forests. However, soil animal diversity and functional community composition differed little between forest types, indicating resilience against disturbance and forest land use. Structural equation modelling suggested that despite a significant influence of forest management on resource abundance and quality, the biomass of most soil fauna functional groups was not directly affected by forest management or resource abundance/quality, potentially because microorganisms hamper the propagation of nutrients to higher trophic levels. Instead, detritivore biomass depended heavily on soil pH. Macrofauna decomposers thrived at high pH, whereas mesofauna decomposers benefitted from low soil pH, but also from low biomass of macrofauna decomposers, potentially due to habitat modification by macrofauna decomposers. The strong influence of soil pH shows that decomposer communities are structured predominantly by regional abiotic factors exceeding the role of local biotic factors such as forest type.

**Supplementary Information:**

The online version contains supplementary material available at 10.1007/s00442-021-04910-1.

## Introduction

Virtually all forests in Central Europe are managed and their natural growth dynamics and overall structure are, therefore, altered (MCPFE 2007; Fischer et al. 2010). This has been shown to reduce aboveground biodiversity (Bengtsson et al. 2000), which is primarily due to a reduction of species negatively affected by higher canopy closure, lower availability of deadwood, and reduced presence of old trees (Humphrey et al. 1999; Grove 2002; Paillet et al. 2010). Despite above- and belowground communities are intrinsically linked, the effect of forest management on the belowground system may differ from that aboveground, as both systems operate at differential temporal and spatial scales (van der Putten et al. 2009). This also implies differential impacts on the structure and functioning of soil animal communities, which are an important component of terrestrial ecosystems, e.g., due to their role in decomposition processes and soil formation (Wardle 2002; Bardgett 2005; Hättenschwiler and Gasser 2005). Soil animal communities have been shown to be useful indicators of forest condition and disturbance (Bird et al. 2000; Ponge et al. 2003; Cassagne et al. 2006). However, effects of forest management vary depending on forest type, intensity of disturbances, and the organism group in focus (Siira-Pietikäinen et al. 2001; Chauvat et al. 2003, 2011). Further, the effect of forest management on soil animal communities may depend on environmental factors, and for understanding the underlying mechanisms interactions between forest management and environmental factors need to be considered.

Important driving factors of soil animal communities varying at regional and local scales include parent rock, precipitation, temperature, soil pH, and soil organic matter content. Their contribution to variations in species compositions of soil animal communities with forest management has been explored in detail for communities of oribatid mites and springtails (Erdmann et al. 2012; Pollierer and Scheu 2017; Russell and Gergócs 2019). Soil pH varying with both parent rock material and stand type fundamentally affects the availability and structure of basal resources of soil food webs, e.g., by changing the species composition of the microbial community (Ruess et al. 1996; Lauber et al. 2008; Rousk et al. 2010; Pollierer et al. 2015), altering the fungi-to-bacteria ratio (Blagodatskaya and Anderson 1998; Högberg et al. 2007; Rousk et al. 2010), and mediating effects of leaf litter stoichiometry on soil fauna (Ott et al. 2014). Acidic and base rich soils feature distinctively different communities of decomposer animals, with macrofauna decomposers, such as earthworms, diplopods, and isopods, reaching highest densities in calcareous soils, whereas mesofauna decomposers, such as Oribatida and Collembola, are dominating in acidic soils (Schaefer and Schauermann 1990; Salmon et al. 2006). Macrofauna decomposers process large amounts of leaf litter (Curry and Schmidt 2007; Melvin and Goodale 2013), whereas most mesofauna decomposers feed on leaf litter-associated fungi and bacteria, thereby translocating litter-derived nutrients into the soil, but contribute little to the degradation of litter material (Chamberlain et al. 2006; Kampichler and Bruckner 2009). As a consequence, the functional composition of the decomposer community may influence the turnover of organic matter and the nutrient status of soils (Schaefer and Schauermann 1990; Hättenschwiler et al. 2005; García-Palacios et al. 2016). However, the turnover of organic matter itself can be an important factor influencing the composition of soil animal communities (Berg and Bengtsson 2007) and may be related to forest management (Bernier and Gillet 2012; Erdmann et al. 2012). Macrofauna decomposers thereby may act as keystone species; by processing leaf litter material reducing the availability of habitat structure and resources for other soil animals, especially litter inhabiting mesofauna (Hättenschwiler et al. 2005; Eisenhauer 2010). However, both trophic and non-trophic interactions between soil animal species, size groups, and trophic groups vary with environmental factors.

Although soil animal communities have been shown to vary with forest type, the characterization into broad management types may not sufficiently capture the underlying drivers (Felipe-Lucia et al. 2018; Penone et al. 2018). Different indices have been developed to assess forest management in more comprehensive and quantitative ways; for instance Schall and Ammer (2013) developed a silvicultural management intensity indicator (SMI), which accounts for tree species, stand age, and different forms of aboveground wooden biomass, including a risk and density component. The Forest Management Intensity Index (ForMI; Kahl and Bauhus 2014) is composed of the proportion of harvested tree volume, the proportion of tree species that are not part of the natural community, and the proportion of deadwood showing signs of saw cuts. Overall, it is similar to the SMI, but easier to assess as it does not include additional assumptions such as estimation of maximum stand carrying capacity or risk potential. The SSC index (SSCI) describes stand structural complexity and is based on terrestrial laser scans of forests. It well explains microclimatic fluctuations in forests and may also be used to explain effects of forest management on soil fauna.

To contribute to the understanding of these interdependencies and to gain insight into effects of forest management on the belowground system, we investigated soil animal communities at high taxonomic resolution over a replicated design spanning four different forest types in each of three different regions. Additionally, a comprehensive set of environmental variables was measured at each study site. We analyzed how the diversity, species composition, and functional structure of soil animal communities are influenced by forest type/management, and evaluated the underlying mechanisms using a structural equation modelling (SEM) approach. SEMs can be applied to observational data in ecological studies and provide a multivariate test of direct and indirect effects and hypothesized causal relationships among multiple correlated variables (Eisenhauer et al. 2015; Fan et al. 2016).

We expected that soil animal communities differ between forest types/management; i.e., we hypothesized that (1) disturbance and habitat modification associated with forest management detrimentally affect the biodiversity of soil animals, resulting in highest species numbers in unmanaged beech forests. Further, we hypothesized that (2) the functional structure of soil animal communities differs between forest types, with low number and biomass of large (macrofauna) decomposers in coniferous and young managed beech forests, due to low nutritional quality of the leaf litter resource and increased disturbance of the microbial community. Finally, we hypothesized that (3) the number and biomass of small (mesofauna) decomposers and associated predators are highest in coniferous forests due to thick leaf litter layers providing ample habitat for mesofauna species.

## Materials and methods

### Study sites

Four replicates of four forest types representing different forest management intensity were sampled in each of three regions of Germany, i.e., Swabian Alb, Hainich-Dün (Hainich), and Schorfheide-Chorin (Schorfheide). The forest types included coniferous forests (Norway spruce (*Picea abies*) in the Swabian Alb and Hainich, and Scots pine (*Pinus sylvestris*) in the Schorfheide), age class stands of young beech (*Fagus sylvatica*) with an approximate age of 30 years (young managed beech), mature age class stands of beech with an age of approximately 70 years (old managed beech), and mature beech stands which have been left unmanaged for approximately 120 years (unmanaged beech). Compared to the natural vegetation of deciduous trees in Central Europe (predominantly beech), coniferous forests represent the most intensively managed forest type, followed by the beech stands in the order young managed beech, old managed beech, and unmanaged beech. The study sites form part of the “Biodiversity Exploratories”, a large integrative biodiversity research project in Germany (www.biodiversity-exploraties.de). The three regions differ in geology and altitude; the Schorfheide is located in a glacial landscape in the north–east of Germany (3–140 m a.s.l.), the Hainich is located in the moderately hilly landscape of Central Germany (285–550 m a.s.l.), and the Swabian Alb in the low mountain range of south–western Germany (480–860 m). Swabian Alb and Hainich both feature calcareous bedrock and soils with high clay content, whereas the soils in the Schorfheide range from sandy loam to almost pure sand (Fischer et al. 2010).

### Sampling and extraction of soil animals

In spring 2008, two large (20 cm diameter) and two small soil cores (5 cm diameter) were taken at random from a 5 m × 5 m subplot on each site. Soil animals from the organic layer and from the top 5 cm of soil were extracted by heat (Macfadyen 1961; Kempson et al. 1963). We recorded abundance and species composition of Araneae, Chilopoda, Coleoptera, Diplopoda, Diplura, Isopoda, Pseudoscorpiones, and Symphyla using large soil cores, while Collembola, Oribatida, and Mesostigmata were analyzed from small soil cores. Lumbricidae were extracted from each site using mustard solution (Gunn 1992; Eisenhauer et al. 2008). The solution was prepared by mixing 100 mg of mustard powder (Semen Sinapis plv., Caesar & Loretz GmbH, Hilden, Germany) with 10 l of water. The mixture was left to steep overnight. At each plot an area of 50 cm × 50 cm was confined using a steel frame, leaf litter was removed and hand sorted for Lumbricidae, and then 5 l of mustard solution was applied to the soil surface. Lumbricidae were collected during the following 15 min, then another 5 l of solution was applied, and Lumbricidae were collected for another 15 min. To include large mobile soil animals, surface active macrofauna and Gastropoda were collected in spring 2011 from the same subplots as the ones sampled in 2008. On each subplot four randomly selected 50 cm × 50 cm areas were confined using a steel frame to prevent mobile animals from escaping. Leaf litter material in the confined area was sieved (1 cm mesh) over plastic trays and animals were collected. Specimens were stored in 70% ethanol until determination. Species were identified using the following keys: Beier (1963), Eason (1964), Freude et al. (1964–2004), Klausnitzer (1978), Gisin (1984), Karg (1989), Klausnitzer (1991–2001), Bogon (1990), Heimer and Nentwig (1991), Hopkin (1991), Karg (1993), Weigmann (2006), Hopkin (2007), Bährmann (2008), and Schaefer (2010).

Species abundances and species numbers of all subsamples were summed up, mean abundances per square meter were calculated for species sampled by litter sieving and heat extraction of soil cores. For the calculation of the biomass of soil animal species and functional groups, either individual specimens were weighed (Lumbricidae), or body lengths were measured (all other macrofauna taxa) or extracted from literature (mesofauna taxa), and body masses calculated via mass-length regressions (Ehnes et al. 2011). For a complete list of species and their affiliation to functional groups, see Appendix S1, Table S1 in supporting information.

### Assessment of environmental factors

The amount of leaf litter in the litter layer was determined by weighing the leaf material of each large soil core after animal extraction. Soil pH was measured in 0.01 M CaCl_2_ solution. C-to-N ratios of leaf litter and fine roots from macrofauna soil cores were measured using an elemental analyzer (NA 1500, Carlo Erba, Milan, Italy). Microbial biomass in leaf litter and soil was assessed by measuring the maximum initial respiratory response (MIRR; mg O_2_ g^−1^ h^−1^) after glucose addition (SIR method; Anderson and Domsch 1978; Beck et al. 1997) in an automated O_2_ micro-compensation apparatus (Scheu 1992). Glucose (80 and 10 mg g^−1^ dry weight for litter and soil, respectively) was added as an aqueous solution to approximately 1 g of leaf litter material adjusting the water content to 80–90% of the water holding capacity (Beck et al. 1997; Joergensen and Scheu 1999). The fungal-to-bacterial ratio as calculated from the relative abundance of fungal and bacterial biomarker phospholipid fatty acids was taken from Pollierer et al. (2015).

### Data analysis

Canonical correspondence analysis (CCA) was applied to analyze the response of species to the environmental factors differing between regions and forest types using CANOCO 4.5 (Jongman et al. 1995; ter Braak and Šmilauer 2002). In this constrained analysis, only the variation accounted for by the environmental factors is used for ordination. To choose the environmental variables that best explained the distribution of species, we used a stepwise forward selection method for pH in soil, litter mass, C-to-N ratios of leaf litter, roots and soil, microbial biomass in leaf litter and soil, and the fungal-to-bacterial ratio in leaf litter (ter Braak and Verdonschot 1995). In the stepwise selection process, microbial biomass in leaf litter and soil, and the C-to-N ratio of roots were dropped due to low explanatory power (*p* > 0.1). The analysis was restricted to species present at a minimum of three sites, i.e., 289 out of 562 species were included. The forest types of each region were coded as supplementary variables not affecting the ordination.

For further statistical analyses all species were assigned to functional groups according to body size (macro- and mesofauna; Swift et al. 1979; Schaefer and Schauermann 1990) and feeding type (decomposers, herbivores, predators) based on literature data and available stable isotope values (Klarner et al. 2014). Mesofauna included taxonomic groups typically not exceeding 1–2 mm in body length as adults (Collembola, Oribatida, Mesostigmata), macrofauna included taxonomic groups of larger body size. “Decomposers” included microbi-detritivorous species predominantly feeding on plant detritus and associated microorganisms. See Appendix S1, Table S1 for a list of all species and their assigned functional groups. Prior to analysis data were inspected for heteroscedasticity using Levene test and log_10_-transformed if necessary to improve homogeneity of variances. Mean values and standard deviation in text and figures are based on non-transformed values.

Multivariate analysis of variance was applied using the datasets for species number, abundances, and biomasses of functional groups to inspect for effects of forest type and region on these variables. To account for variance caused by regional differences, region was included as random effect in univariate analyses analyzing the effect of forest type on the above mentioned response variables using linear mixed effects models (package ‘lme4′ and ‘lmerTest’). Similar analyses were applied to investigate the effect of forest type on environmental factors. In case of significant differences in univariate analyses, Tukey’s honestly significant difference test was used to inspect differences between means using the R package ‘multcompView’ (Graves et al. 2015). Results of multivariate analyses are reported as mean ± sd.

Pearson correlations within regions were used to analyze the interrelation of log-transformed soil animal biomasses as influenced by environmental factors. As sample size was equal in all regions (*n* = 16) we also report mean correlation coefficients across regions with significances based on one-tailed *t* tests (Appendix S2, Table S1). Based on correlation analysis and on previous knowledge as described in the introduction, an initial path diagram (see Appendix S3, Fig. S1) with hypothesized causal relationships between forest management, environmental variables, and biomasses of meso- and macrofauna functional groups was constructed. We omitted the C-to-N ratio of soil in the analysis as it was significantly correlated with the C-to-N ratio of leaf litter and with the amount of leaf litter. For identifying causal relationships across regions, we implemented structural equation modelling in R using the packages ‘piecewiseSEM’ (Lefcheck 2016), ‘nlme’, ‘lme4’, and ‘lattice’. The package ‘piecewiseSEM’ performs local estimation for each endogenous node and its predictors in the model, allowing to include random slopes and intercepts for the region if needed. After visual inspection of correlations and their interaction with region, we implemented random slopes and intercepts using linear mixed effects models (‘lmer’) and tested whether their inclusion improved the individual model using the function ‘ranova’ from the ‘lmerTest’ package in R (Kuznetsova et al. 2017). If the individual model was not improved (lower AIC if random effect was dropped), the random slopes/intercepts were removed. To quantify forest management, models with the three different indices ForMI, SMI, and SSCI were compared, and the index which resulted in the model with the lowest AIC, i.e., best explained the data, was chosen for the final model (for the full final model, individual R-square values and the Chi-square difference test between models using ForMI, SMI, and SSCI, see Appendix S3, Tables S1, S2, and S3). The dataset did not comprise missing data and multivariate normality was assessed using the Henze–Zirkler's multivariate normality test (R package ‘MVN’; Korkmaz et al. 2014). Data were rescaled to account for different scales of predictor variables. The package ‘piecewiseSEM’ includes the calculation of missing paths in the model and indicates whether they are significant. There was a missing path from the C-to-N ratio of roots to mesofauna predator mass which was indicated to be significant. However, we did not include this path since a direct effect is unlikely. In addition, the path from microbial biomass of litter to microbial biomass of soil was indicated to be significant. We included this path as covariance in the final model. Except for CCA, all statistical analyses were performed using R 4.0.2 (R Development Core Team 2013).

## Results

### Species composition of soil animal communities across regions

Overall 562 species of soil animals were identified. The most species rich groups were macrofauna predators (179 species), followed by mesofauna decomposers (169 species), mesofauna predators (121 species), macrofauna decomposers (71 species), and macrofauna herbivores (22 species).

**Fig. 1:**
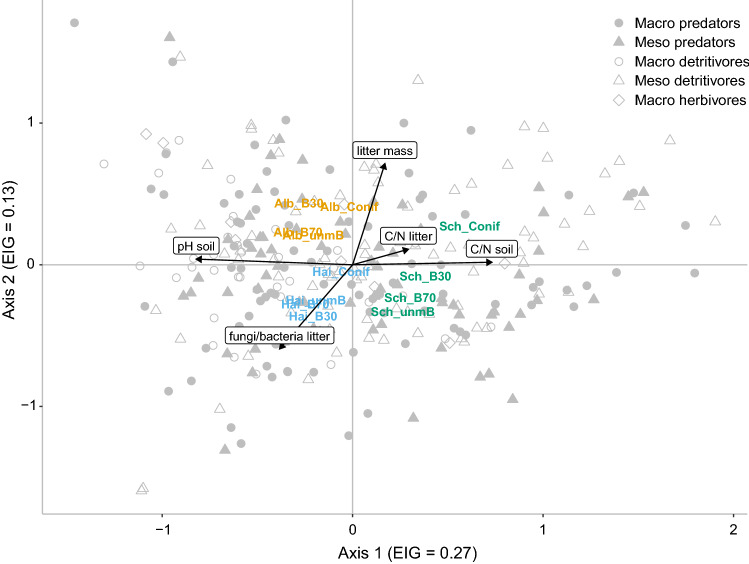
The first two axes of canonical correspondence analysis (CCA) of soil animal species from different forest types in three regions [Swabian Alb (SA), Hainich (Hai), Schorfheide-Chorin (Sch); Conif = coniferous forest, B30 = young managed beech, B70 = old managed beech, unmB = unmanaged beech] as related to environmental factors chosen by stepwise forward selection [amount of leaf litter (litter mass), C-to-N ratio of litter, soil and fine roots (C/N litter, C/N soil and C/N roots, respectively), soil pH and the fungal-to-bacterial ratio of leaf litter (fungi/bacteria litter)]. Species are marked by symbols indicating functional group affiliation (see legend); environmental factors accounted for 19.2% of variation and the first and second axes cumulatively explained 8.6% and 12.7% of variation, respectively

Canonical correspondence analysis of soil animal species (Fig. 1) separated forest types of the Schorfheide from those of the Swabian Alb and Hainich along the first axis. The sites correlated with soil pH (contribution 29.8%, pseudo-F = 3.8, *p* = 0.002) corresponding to alkaline conditions in the calcareous soils of the Swabian Alb and Hainich compared to acidic soils with high C-to-N ratios (contribution 12.7%, pseudo-F = 1.7, *p* = 0.004) in soils of the Schorfheide. The second axis separated the Swabian Alb from the Hainich; higher amounts of leaf litter (contribution 14.0%, pseudo-F = 1.8, *p* = 0.002) in the Swabian Alb contrasted higher fungal-to-bacterial ratio in leaf litter (contribution 9.8%, pseudo-F = 1.3, *p* = 0.028) in the Hainich. Further, the second axis separated the coniferous forests from beech forests in the Schorfheide and Hainich with the fungal-to-bacterial ratio and the amount, and to a lesser extent the C-to-N ratio of leaf litter (contribution 9.1%, pseudo-F = 1.2, *p* = 0.08) contributing to this separation. The analysis further reflected that a similar number of species of macrofauna predators and both functional groups of mesofauna were associated with the three regions; by contrast, most species of macrofauna herbivores and decomposers were scarce in coniferous forests, in particular in the Schorfheide.

### Characteristics of the soil and litter habitat

**Fig. 2:**
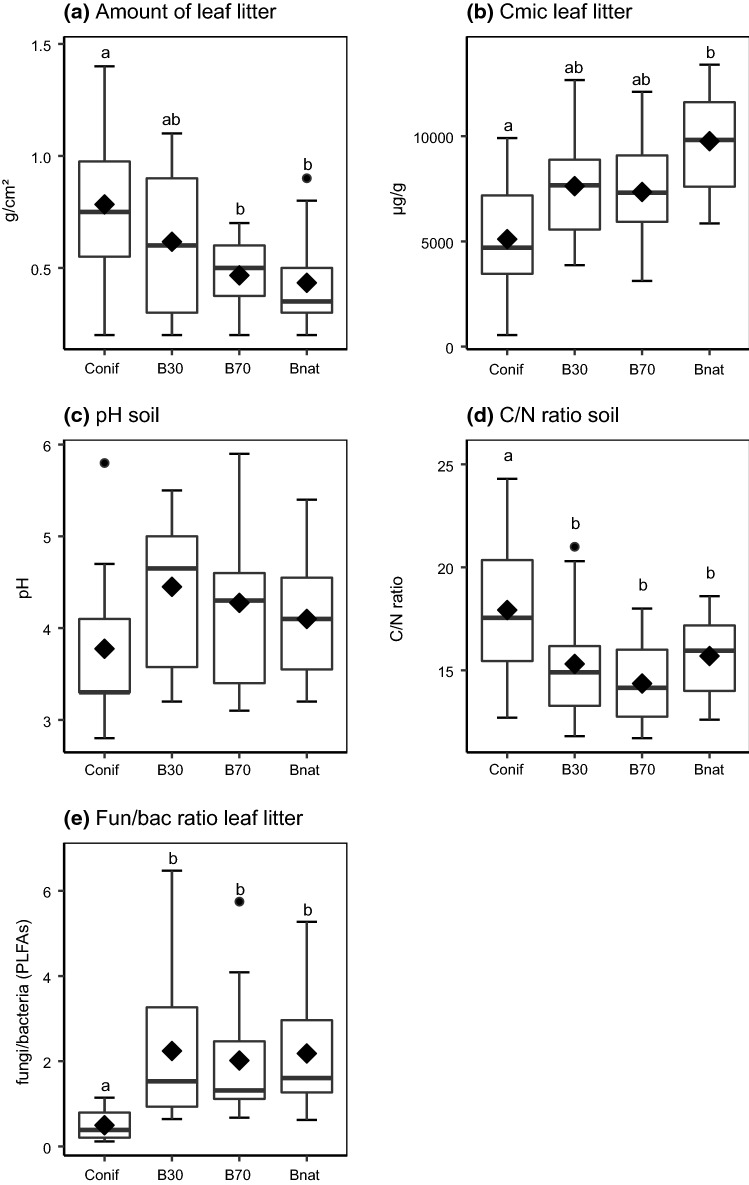
Amount of leaf litter (**a**), microbial biomass concentration in leaf litter (*C*_mic_) (**b**), soil pH (**c**), C-to-N (C/N) ratio of soil and fungal-to-bacterial (fun/bac) ratio of leaf litter in coniferous forests (Conif), and in young (B30), old (B70), and natural beech forests (Bnat). Boxes indicate the 25th and 75th‰, the line in the box marks the median, whiskers map the 90th and 10th‰, dots display outliers, diamonds indicate the mean; different letters indicate significant differences between means (p < 0.05, Tukey’s HSD). For statistical analysis see Table 1

After accounting for regional variations, the environmental factors investigated differed significantly between forest types (Table 1). Amount of leaf litter decreased from coniferous to young managed beech to old managed to unmanaged beech forests (Fig. 2a). Concentration of microorganisms (*C*_mic_) in leaf litter increased from coniferous to young and old managed beech to unmanaged beech forests (Fig. 2b). Univariate analysis indicated significant differences in soil pH between forest types (Fig. 2c). The C-to-N ratio of soil was significantly higher in coniferous compared to beech forests, whereas the fungal-to-bacterial ratio of leaf litter was significantly lower in coniferous forests than in beech forests (Figs. 2d, e). Concentrations of microorganism in soil (*C*_mic_) and C-to-N ratios of leaf litter and fine roots did not differ significantly between forest types (Table 1). Means and standard deviation of each of the environmental factors studied in the different regions and forest types are given in Appendix S4, Table S1 (Supplementary material).

**Table 1:** Results of multi- and univariate analyses of variance on the effect of forest type and region on the environmental variables studied (*C*_mic_ = microbial biomass, C/N = C-to-N ratio, fun/bac = fungal-to-bacterial ratio); univariate effects were tested with linear mixed effects models using region as random effect; num *df* numerator degrees of freedon, *den df* denominator degrees of freedom; significant differences are highlighted in bold, *p < 0.05, **p < 0.01, ***p < 0.001

	Factor	Wilk's λ	num *df*	den *df*	*F* value
Multivariate analysis	Forest type	0.21	24	102.11	3.05***
	Region	0.14	16	70.00	7.31***
Univariate analysis					
Amount of leaf litter	Forest type		3	42	4.60**
*C*_mic_ soil	Forest type		3	42	2.04
*C*_mic_ leaf litter	Forest type		3	42	6.83***
Soil pH	Forest type		3	42	3.56*
C/N leaf litter	Forest type		3	42	0.69
C/N fine roots	Forest type		3	42	2.42
C/N soil	Forest type		3	42	6.30**
fun/bac leaf litter	Forest type		3	42	6.29**

### Diversity and functional structure of the soil animal community

**Fig. 3:**
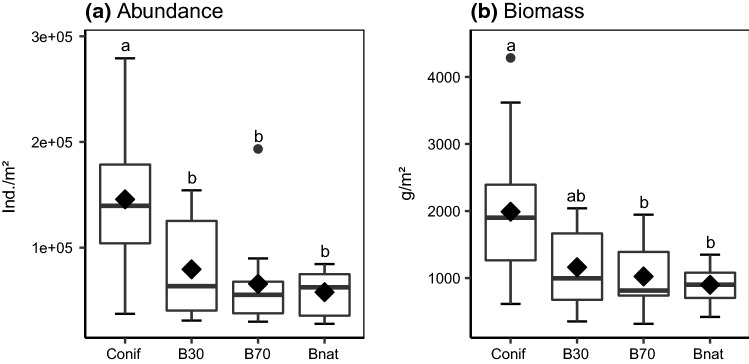
Abundance (**a**) and biomass (**b**) of mesofauna decomposers in different forest types (Conif = coniferous forest, B30 = beech age class 30, B70 = beech age class 70, Bnat = unmanaged beech); boxes indicate the 25th and 75th‰, the line in the box marks the median, whiskers map the 90th and 10th‰, dots display outliers, diamonds indicate the mean; different letters indicate significant differences between means (*p* < 0.05, Tukey’s HSD)

Species number of soil animals differed significantly between forest types after accounting for regional variations (Table 2). Macrofauna herbivores were more diverse in unmanaged (3.9 ± 1.6 species/m^2^) compared to old managed beech forests (1.9 ± 1.1 species/m^2^); values in young managed beech and coniferous forests were intermediate (Appendix S4, Table S3). In trend (*p* = 0.054), the diversity of mesofauna decomposers was higher in coniferous forests (26.7 ± 5.6 species/m^2^) than in unmanaged beech forests (21.6 ± 4.0 species/m^2^), with intermediate values in young and old managed beech (Appendix S4, Table S3). Species number of other soil animal functional groups did not differ significantly between forest types (Table 2). For species numbers of the other soil animal functional groups in the different regions and forest types investigated see Table S2, Appendix S4. Density and biomass of soil animals also differed significantly between forest types (Table 2). Mesofauna decomposers had significantly higher densities in coniferous forests compared to the three types of beech forest investigated (Fig. 3a). Their biomass followed a similar pattern, decreasing from coniferous forests to young managed beech to old managed and unmanaged beech forests (Fig. 3b). Density and biomass of the other functional groups investigated did not differ significantly between forest types (Table 2). For mean density and biomass of soil animal functional groups in the different regions and forest types investigated see Appendix S4, Tables S2 and S4, respectively.

**Table 2:** Results of multi- and univariate analyses of variance on the effect of forest type on number of species, density and biomass of different soil animal functional groups; region was included as block; significant differences are highlighted in bold, *p < 0.05, **p < 0.01, ***p < 0.001

	Factor	Species number				Abundance				Biomass			
		Wilk's λ	num df	den df	*F* value	Wilk's λ	num df	den df	*F* value	Wilk's λ	num df	den df	*F* value
Multivariate analysis	Region	0.34	10	76.0	5.49***	0.52	10	76.0	2.96**	0.51	10	76.0	3.01**
	Forest type	0.53	15	105.3	1.83*	0.45	15	105.3	2.38**	0.37	15	105.3	3.03***

Univariate analysis													
Functional group	Factor	Species number				Abundance				Biomass			
Macrofauna detritivores	Forest type		3	42	1.95		3	42	0.91		3	42	0.25
Macrofauna herbivores	Forest type		3	42	3.82*		3	42	1.87		3	42	1.81
Macrofauna predators	Forest type		3	42	1.65		3	42	1.52		3	42	2.50
Mesofauna detritivores	Forest type		3	42	2.75		3	42	9.81***		3	42	7.60**
Mesofauna predators	Forest type		3	42	1.40		3	42	1.70		3	42	1.60

### Functional structure of soil animal communities as affected by environmental factors and interrelations between functional groups

With few exceptions, correlations between soil animal biomasses and environmental factors followed similar trends in the three regions. Macrofauna decomposer biomass increased with soil microbial biomass, soil pH and the fungal-to-bacterial ratio of leaf litter and was negatively correlated with the mass of leaf litter and in the Schorfheide with the C-to-N ratio of leaf litter (Appendix S2, Fig. S1, Table S1). The biomass of mesofauna decomposers increased with the mass of leaf litter and with increasing forest management as indicated by ForMI and SMI; it decreased with soil pH, with the fungal-to-bacterial ratio of leaf litter and with soil microbial biomass, whereas the correlation with litter microbial biomass was negative in the Swabian Alb and Schorfheide, but positive in the Hainich. Mesofauna predator biomass increased with the C-to-N ratio of fine roots and soil, but decreased with soil pH; however, effects were not significant in the Schorfheide. The biomass of mesofauna decomposers and mesofauna predators was correlated significantly in the Hainich and Schorfheide; furthermore, the biomass of both groups decreased with the biomass of macrofauna decomposers, in particular in the Hainich. The ForMI was negatively correlated with microbial biomass and the fungal-to-bacterial ratio of leaf litter, and with the biomass of macrofauna predators. It was positively correlated with litter mass and the biomass of mesofauna detritivores. Correlations of the SMI with environmental variables and biomasses of soil animal groups were similar, whereas the SSC index was correlated with fewer environmental variables (positively with microbial biomass in leaf litter and soil, and negatively with the C-to-N ratio of soil), and only significantly positively correlated with the biomass of macrofauna detritivores. ForMI and SMI were significantly positively correlated, whereas SSC was negatively correlated with ForMI.

**Fig. 4:**
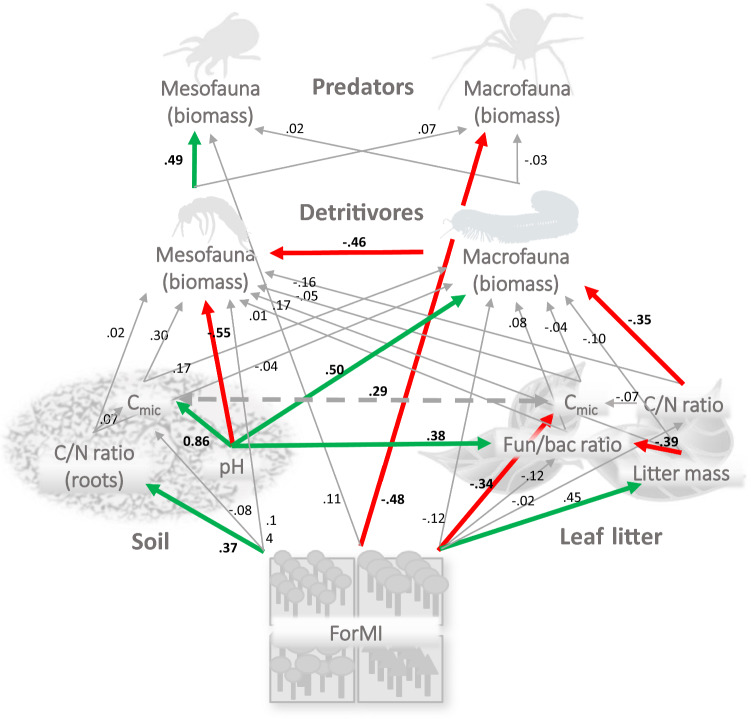
Path diagram showing the structural equation model of the hypothesized relationship between forest management intensity (ForMI), characteristics of leaf litter and soil [leaf litter and soil microbial biomass (C_mic_), litter mass, C-to-N (C/N) ratio of leaf litter and roots, fungal-to-bacterial (Fun/bac) ratio in litter, and soil pH], and the biomass of meso- and macrofauna detritivores as well as meso- and macrofauna predators. The data did not significantly deviate from the model (Fisher’s *C* = 63.56, *P* = 0.35, 60 degrees of freedom). Colored arrows denote significant (*P* < 0.05) positive (green) or negative (red) effects, thin grey arrows represent non-significant effects (*P* > 0.1); the direction of the arrows indicates the hypothesized direction of effects. The double-headed dashed arrow indicates a significant correlation. Numbers on arrows indicate standardized path coefficients

According to piecewise SEM analysis (Fig. 4), forest management intensity as represented by the ForMI influenced the amount and microbial biomass in leaf litter and the C-to-N ratio of roots (for unstandardized and standardized regression weights and significance levels see Table 3). Although microbial biomass in leaf litter and soil, the fungal-to-bacterial ratio of leaf litter and the amount of leaf litter were (in part) significantly correlated with the biomass of each detritivores and predators, SEM analysis indicated that these parameters were not the main drivers of the biomass of soil animals. Instead, pH strongly influenced decomposer biomass, with macrofauna decomposers thriving at high pH and mesofauna decomposers at low pH. High pH also positively influenced the fungal-to-bacterial ratio in leaf litter and the microbial biomass in soil, with the latter being significantly correlated with the microbial biomass in leaf litter. Microbial parameters, however, had no significant effects on soil animal functional groups. The C-to-N ratio of leaf litter was not affected by forest management intensity, but it had a direct negative effect on the biomass of macrofauna detritivores. In addition to differential effects of soil pH, macrofauna detritivores also had a direct negative influence on the biomass of mesofauna detritivores. The only soil animal functional group that was directly affected by ForMI were macrofauna predators, which decreased in biomass with increasing land-use intensity. The biomass of mesofauna predators was positively influenced by the biomass of their potential prey, i.e., mesofauna decomposers, whereas the biomass of macrofauna predators neither depended on the biomass of mesofauna decomposers nor on that of macrofauna decomposers.

**Table 3:** Standardized and unstandardized coefficients (estimates), standard errors (Std.Error), degrees of freedom (df), critical values (Crit.Value) and *p* values (P.Value) for the effect of predictors (CN litter—C-to-N ratio of leaf litter, CN roots—C-to-N ratio of roots, Cmic litter—microbial biomass in leaf litter, Cmic soil—microbial biomass in soil, ForMI—index of forest management intensity, Funbac ratio litter—fungal-to-bacterial ratio in leaf litter, Litter mass—mass of leaf litter, Macro detritivores—biomass of macrofauna detritivores, Meso detritivores—biomass of mesofauna detritivores and Soil pH—pH in soil) on response variables including in addition the biomass of mesofauna and macrofauna predators (meso predators and macro predators, respectively) in the final piecewise SEM model (see Fig. 4); significant effects are highlighted in bold, *p < 0.05, **p < 0.01, ***p < 0.001

Response	Predictor	Estimate	Std.Error	df	Crit.Value	P.Value	Std Estimate	
Meso predators	Macro detritivores	0.01	0.06	44	0.14	0.89	0.02	
**Meso predators**	**Meso detritivores**	**0.53**	**0.20**	**44**	**2.66**	**0.01**	**0.49**	*
Meso predators	ForMI	0.05	0.05	44	0.84	0.41	0.11	
Macro predators	Macro detritivores	− 0.01	0.07	44	− 0.14	0.89	− 0.03	
**Macro predators**	**ForMI**	**− 0.22**	**0.06**	**44**	**− 3.40**	**0.00**	**− 0.48**	**
Macro predators	Meso detritivores	0.09	0.23	44	0.37	0.71	0.07	
**Meso detritivores**	**Macro detritivores**	**− 0.13**	**0.05**	**38**	**− 2.56**	**0.01**	**− 0.46**	*
Meso detritivores	ForMI	0.05	0.05	38	1.10	0.28	0.14	
**Meso detritivores**	**Soil pH**	**− 0.17**	**0.07**	**38**	**− 2.48**	**0.02**	**− 0.55**	*
Meso detritivores	CN litter	− 0.01	0.01	38	− 1.32	0.19	− 0.16	
Meso detritivores	Cmic litter	0.00	0	38	− 0.47	0.64	− 0.05	
Meso detritivores	Litter mass	0.13	0.10	38	1.30	0.20	0.17	
Meso detritivores	Cmic soil	0.00	1e− 04	38	1.50	0.14	0.30	
Meso detritivores	CN roots	0.00	0.01	38	0.20	0.84	0.02	
Meso detritivores	Funbac ratio litter	0.00	0.02	38	0.10	0.92	0.01	
Macro detritivores	ForMI	− 0.15	0.15	39	− 1.03	0.31	− 0.12	
Macro detritivores	Litter mass	− 0.29	0.31	39	− 0.91	0.37	− 0.10	
Macro detritivores	Cmic litter	0.00	0	39	− 0.36	0.72	− 0.04	
**Macro detritivores**	**CN litter**	**− 0.05**	**0.01**	**39**	**− 3.80**	**0.00**	**− 0.35**	***
**Macro detritivores**	**Soil pH**	**0.52**	**0.19**	**39**	**2.69**	**0.01**	**0.50**	*
Macro detritivores	Cmic soil	0.00	3e− 04	39	0.95	0.35	0.17	
Macro detritivores	CN roots	− 0.01	0.02	39	− 0.41	0.69	− 0.04	
Macro detritivores	Funbac ratio litter	0.04	0.07	39	0.64	0.52	0.08	
**Cmic litter**	**ForMI**	**− 1529.46**	**639.76**	**45**	**− 2.39**	**0.02**	**− 0.34**	*
Cmic litter	CN litter	− 39.61	78.37	45	− 0.51	0.62	− 0.07	
**Litter mass**	**ForMI**	**0.21**	**0.06**	**46**	**3.44**	**0.00**	**0.45**	**
CN litter	ForMI	− 0.14	2.62	2	0.00	0.96	− 0.02	
Cmic soil	CN roots	6.99	9.53	44	0.73	0.47	0.07	
**Cmic soil**	**Soil pH**	**537.23**	**52.11**	**44**	**10.31**	**0.00**	**0.8**	***
Cmic soil	ForMI	− 59.98	65.93	44	− 0.91	0.37	− 0.08	
**CN roots**	**ForMI**	**2.63**	**0.97**	**46**	**2.70**	**0.01**	**0.37**	**
Funbac ratio litter	ForMI	− 0.28	0.30	44	− 0.92	0.36	− 0.12	
**Funbac ratio litter**	**Litter mass**	**− 1.92**	**0.67**	**44**	**− 2.89**	**0.01**	**− 0.39**	**
**Funbac ratio litter**	**Soil pH**	**0.71**	**0.22**	**44**	**3.16**	**0.00**	**0.38**	**
** ~ ~ Cmic litter**	** ~ ~ Cmic soil**	**0.29**	**− **	**48**	**2.05**	**0.02**	**0.29**	*

## Discussion

We investigated diversity, density, and biomass of soil animal communities at species and functional group level in differently managed forests across three regions in Germany. Major abiotic and biotic environmental variables were recorded and used to explain changes in soil animal communities with forest management intensity across regions. We demonstrated that although leaf litter and soil biotic attributes are strongly influenced by different forest types and by the intensity of forest management, they only play a subordinate role in explaining the biomass of soil meso- and macrofauna detritivores. Instead, pH exerts a major influence on the biomass of detritivores, suggesting that they are mainly affected by regional abiotic factors such as parent rock and, therefore, may be buffered against changes in biotic conditions caused by forest management. While the biomass of mesofauna predators mainly depended on the biomass of their potential prey, i.e., mesofauna decomposers, macrofauna predators were the only group that was directly negatively affected by the intensity of forest management, suggesting that they suffer from disturbance and reduced habitat complexity.

### The structure of the soil animal community as influenced by regional factors and forest type

Species composition and abundance of soil animal communities was mainly influenced by regional factors, whereas communities of different forest types were only separated within regions in CCA. This suggests that the importance of regional abiotic factors, such as temperature, precipitation, and parent rock, presumably surpasses that of different forest types as structuring force of soil animal communities as indicated earlier for certain soil animal groups (Erdmann et al. 2012; Pollierer and Scheu 2017). Notably, the same has been found for microbial community structure (Pollierer et al. 2015; Richter et al. 2018). Regional factors were particularly important in explaining abundance and species composition of macrofauna herbivore and decomposer communities, thriving at the Hainich and Swabian Alb, regions with high soil pH, while the opposite was true for the Schorfheide. Differences in soil animal communities between forest types were mainly explained by the amount of leaf litter and the fungal-to-bacterial ratio of leaf litter, illustrating the changes in biotic conditions associated with forest management. CCA further showed that species composition of coniferous forests differed most from that of unmanaged beech forests in each of the three regions investigated, indicating that habitat conditions in coniferous forests deviate most from those of the presumed natural forests of central Europe dominated by beech.

After accounting for regional variability, the analysis of environmental factors supports the assumption that the habitat of soil animals is significantly influenced by forest management, i.e., by anthropogenic disturbances. The thickness of the leaf litter layer decreased from coniferous forests to young managed beech to mature beech forests. Notably, the opposite was true for microbial biomass concentration and the fungal-to-bacterial ratio in leaf litter, which increased from coniferous forests to unmanaged beech forests. Presumably, this is related to significantly lower C-to-N ratios and higher pH in soils of beech compared to coniferous forests. Despite these pronounced differences in biotic and abiotic conditions, and contrasting our hypothesis, the biodiversity and functional composition of the soil animal community was little affected by forest type. As Penone et al. (2018) suggested, specific forest features may be better at explaining variations in animal communities than forest types. Macrofauna herbivores, the least abundant functional group investigated, were the only functional group of soil animals that differed in species numbers between forest types. Macrofauna herbivores were more diverse in unmanaged compared to old managed beech forests, indicating that management reduces the number of niches for plant feeding arthropods in old-growth beech forests. Interestingly, plant diversity at our study sites has been shown to increase with management of old beech stands (Boch et al. 2013). This indicates that the number of trophic niches of soil- and litter-dwelling herbivores is unlikely to be affected directly by the aboveground diversity of plants. The animals studied included a large number of root-feeding species, such as curculionid and elaterid beetle larvae, suggesting that natural beech forests including differently aged trees provide a higher number of niches for root feeders than age class managed beech forests. Despite no significant differences in species numbers, the abundance and biomass of mesofauna detritivores differed significantly between forest types, with both higher abundance and biomass in coniferous compared to beech forests. Thick leaf litter layers in coniferous forests may favor the abundance of mesofauna by increasing the available habitat (Erdmann et al. 2012), whereas higher C-to-N ratios and lower microbial biomass of leaf litter in coniferous forests had no detrimental effect on the abundance and biomass of mesofauna detritivores.

### Relationships between environmental factors and biomass of functional groups of soil animals

The piecewise structural equation modelling approach allowed to identify major drivers for the biomass of soil animal functional groups apart from regional differences, which were included in the model as random effects. The SEM analysis suggested that forest management, as represented by the ForMI index, strongly influences resource availability of the soil food web by affecting leaf litter mass, and leaf litter and root C-to-N ratios, but also leaf litter microbial biomass. Fungal-to-bacterial ratios were indirectly affected by the altered leaf litter mass. However, this differential availability of basal resources did not exert strong effects on the biomass of detritivores, in particular mesofauna detritivores. This is in line with findings of Klarner et al. (2014) who suggested that nutrients in leaf litter are locked up by microorganisms, hampering their propagation to higher trophic levels. Instead, the biomass of detritivores was strongly influenced by pH, suggesting again that abiotic factors are major determinants of decomposer communities. Litter quality, as indicated by the C-to-N ratio of leaf litter, significantly influenced the biomass of macrofauna decomposers, but not that of mesofauna decomposers, suggesting that the former depend more directly on leaf litter as resource. In nutrient addition experiments, responses to elevated nutrient availability were also mainly confined to macrofauna (Scheu and Schaefer 1998), whereas effects on mesofauna were limited (Maraun et al. 2001). Further, macrofauna detritivores had a direct negative influence on the biomass of mesofauna detritivores, presumably by reducing the availability of habitat structure and resources (Hättenschwiler and Gasser 2005). In addition, this negative effect may be caused by disturbance due to perturbation of leaf litter and soil layers, e.g., by burrowing activities of earthworms (Maraun et al. 2003; Eisenhauer 2010). Since microbial resources and the quality of leaf litter and soil did not significantly influence the biomass of mesofauna decomposers, resource competition presumably plays a minor role for the negative effect of macrofauna on mesofauna biomass.

The only soil animal functional group that was directly affected by forest management intensity were macrofauna predators indicating that they suffer from disturbance and reduced habitat complexity associated with increasing forest management (Potapov et al. 2020). In addition, increasing amounts of deadwood and vertical stand heterogeneity in unmanaged stands may increase prey diversity, potentially supplementing soil predators with additional aboveground prey (Müller et al. 2018; Penone et al. 2018). Also, the higher (root) herbivore diversity in unmanaged forests may provide additional prey for macrofauna predators. By contrast, mesofauna predators depended directly on the biomass of their potential prey, i.e., mesofauna decomposers, suggesting that they are bottom-up controlled. Interestingly, as indicated by the lack of correlation with macrofauna decomposers, mesofauna predators did not suffer from associated disturbances in a similar way as mesofauna decomposers.

Overall, the results indicate that anthropogenic disturbances associated with the management of forests only little affect the structure and functioning of soil animal communities. Characteristic features of soil animal food webs, such as the dominance of generalist feeders and redundancy within functional groups, likely buffer its architecture against disturbances (Siira-Pietikäinen et al. 2001; Scheu 2002; Cole et al. 2006). Furthermore, soil animal communities presumably recover quickly from disturbances associated with forest management practices; indeed, density and diversity of soil mites have been shown to recover within four years after clear cutting and replanting (Hasegawa et al. 2013). Our data suggest that the structure of soil animal communities of young managed, old managed, and unmanaged beech forests is similar within each of the three regions investigated. This supports the view of Swanson et al. (2011) that early successional stages such as young beech forests may conserve a large fraction of the fauna of old-growth forest stands. Results of the study suggest that this even applies to coniferous forests, especially for soil mesofauna. However, the direct negative influence of forest management on macrofauna predators suggests that this functional group responds more sensitively to disturbances. Macrofauna predators may depend in part on aboveground prey (von Berg et al. 2010) which more sensitively responds to forest management than belowground animals (Penone et al. 2018). Loss of macrofauna predators potentially feeds back to lower trophic levels and can even impact leaf litter decomposition (Melguizo-Ruiz et al. 2020).

A shortcoming of the present study is that we did not account for nematodes and enchytraeids in soil, which are a potentially important prey for mesofauna predators. Piecewise SEM suggested a missing positive path from root C-to-N ratios to the biomass of mesofauna predators. We did not include this path as it is unlikely that mesofauna predators would directly feed on roots, but it could be an indication of their dependence on root-feeding nematodes. Presumably, high C-to-N ratios of fine roots are related to high root growth and exudation, and these characteristics are driven by low nitrogen availability (Boxman et al. 1998; Paterson and Sim 2000). Roots and root-colonizing microbes are the main food source for soil nematodes (Bais et al. 2006; Crotty et al. 2011), these in turn are a main prey for predatory microarthropods (Karg 1983; Koehler 1999; Heidemann et al. 2011). Potentially increased root growth and root exudation fosters mesofauna predators via a trophic cascade involving three to four trophic levels.

## Conclusions

Regional variations of environmental factors, in particular those related to parent rock and soil pH, strongly influence the species composition of soil animal communities in managed and unmanaged forests in Central Europe. Locally, however, forest management and forest type affect soil animal communities in particular via changes in environmental factors associated with structural characteristics of the soil and litter habitat. However, diversity, abundance, and in particular biomass distribution of functional groups of soil animals are rather insensitive to changes in forest type/management. This indicates that while individual species may be influenced, the overall structure and functioning of soil animal communities are buffered against anthropogenic disturbances, and ecosystem services provided by soil animals are likely to be maintained even if forests are markedly altered by man. However, to preserve the full complement of soil animal species including rare species and large predators, unmanaged forests are needed. Considering the turnover of species on regional scales such forests need protection to conserve the diversity of soil animal species and their functioning.

## Supplementary Information

Below is the link to the electronic supplementary material.

Supplementary file1 (DOCX 388 kb)

Supplementary file2 (DOCX 1559 kb)

Supplementary file3 (DOCX 28 kb)

Supplementary file4 (DOCX 76 kb)

## Data Availability

All data generated or analyzed during this study are included in this published article and its supplementary information files.
