# Supplementary material for: Diversity and functional structure of soil animal communities suggest soil animal food webs to be buffered against changes in forest land use
Source: Oecologia. 2021 Apr 14;196(1):195–209. doi: 10.1007/s00442-021-04910-1 (PMC8139884; doi:10.1007/s00442-021-04910-1)
Supplement: Supplementary file 1 — Supplementary file1 (DOCX 388 kb) [file 442_2021_4910_MOESM1_ESM.docx]

**Appendix S3**


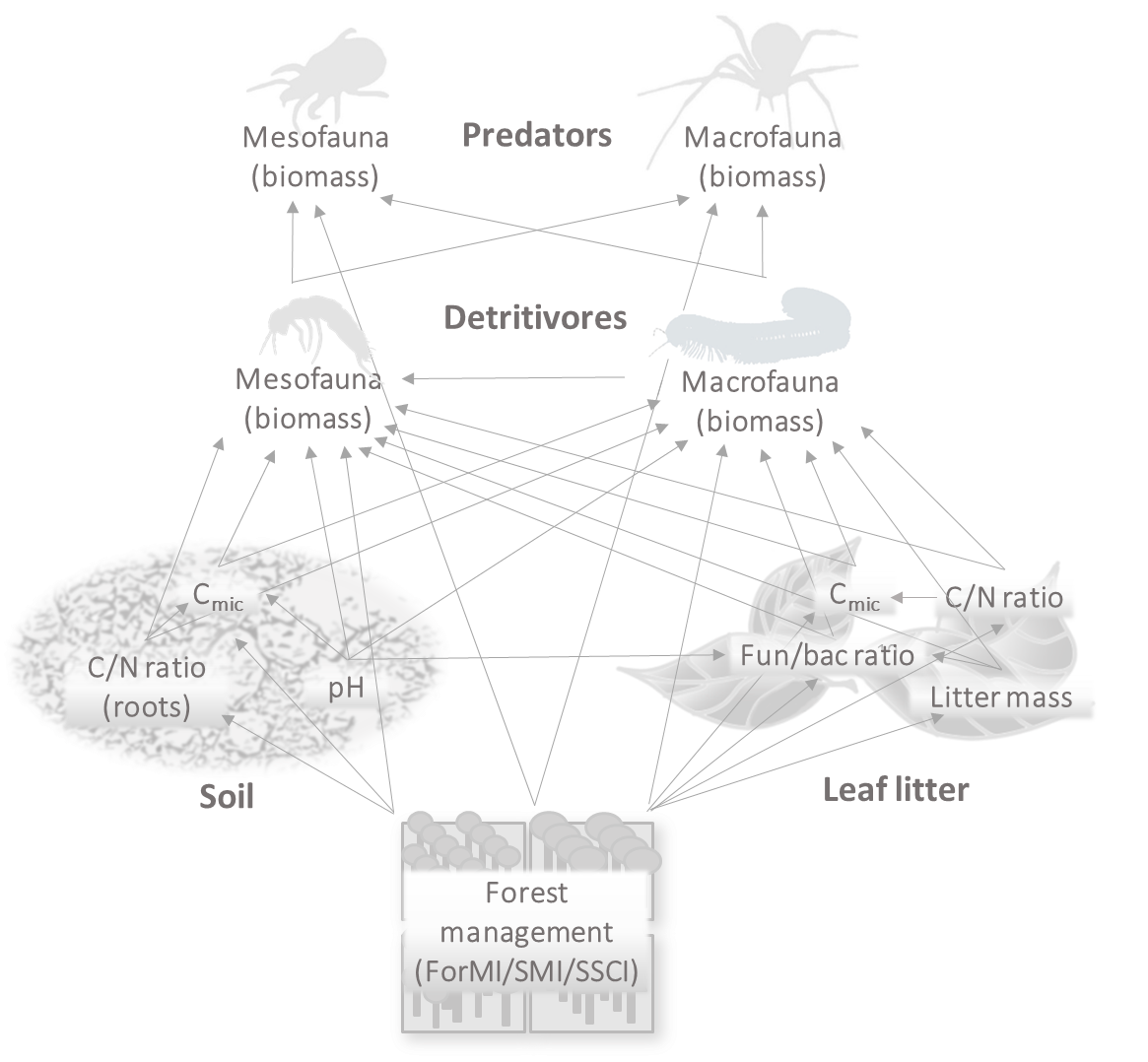


**Fig. S1**: Initial hypothesized causal relationships between forest management as indicated by the Forest Management Intensity Index (ForMI), the silvicultural management intensity indicator (SMI) and the SSC-index (SSCI), environmental parameters (amount of leaf litter – Litter mass, the fungal-to-bacterial ratio of leaf litter – Fun/bac ratio, the C-to-N ratio of leaf litter and roots – C/N ratio, the microbial biomass of leaf litter and soil – Cmic, and soil pH – pH), and the biomass of soil animal functional groups (meso- and macrofauna detritivores and predators).

**Table S1:** Structural equations used in the final piecewise SEM.

| Meso_predators ~ Macro_detritivores+Meso_detritivores+ForMI |
| --- |
| Macro_predators ~ Macro_detritivores+ForMI+Meso_detritivores |
| Meso_detritivores ~ Macro_detritivores+ForMI+soil_pH+CN_litter+Cmic_litter+litter_mass+Cmic_soil+CN_roots  +funbac_ratio_leaflitter |
| Macro_detritivores ~ ForMI+litter_mass+Cmic_litter+CN_litter+soil_pH+Cmic_soil+CN_roots+funbac_ratio_leaflitter |
| Cmic_litter ~ ForMI+CN_litter |
| litter_mass ~ ForMI |
| CN_litter ~ ForMI+(1+ForMI\|Region) |
| Cmic_soil ~ CN_roots+soil_pH+ForMI |
| CN_roots ~ ForMI |
| funbac_ratio_leaflitter ~ ForMI+litter_mass+soil_pH |
| Cmic_litter %~~% Cmic_soil |

**Table S2:** Individual R-squared values for the models presented in Table S1.

| Response | Marginal | Conditional |
| --- | --- | --- |
| Meso_predators | 0.27 | - |
| Macro_predators | 0.21 | - |
| Meso_detritivores | 0.62 | - |
| Macro_detritivores | 0.68 | - |
| Cmic_litter | 0.11 | - |
| litter_mass | 0.20 | - |
| CN_litter | 0.00 | 0.32 |
| Cmic_soil | 0.72 | - |
| CN_roots | 0.14 | - |
| funbac_ratio_leaflitter | 0.39 | - |

**Table S3:** Chi-square difference test between SEM models using the Forest Management Intensity Index (ForMI), the silvicultural management intensity indicator (SMI) and the SSC-index (SSCI) as indicator of forest management. The model using ForMI had a significantly lower AIC and BIC and was therefore best suited as explanatory variable in the model. *** p < 0.0001

|  | AIC | BIC | Fisher.C | Fisher.C.Diff | DF.diff | P.value |  |
| --- | --- | --- | --- | --- | --- | --- | --- |
| ForMI | 179.654 | 286.312 | 65.654 |  |  |  |  |
| vs. SMI | 182.852 | 289.852 | 68.852 | 3.198 | 0 | 0 | *** |
| vs. SSCI | 181.194 | 287.852 | 67.194 | 1.540 | 0 | 0 | *** |
