# Supplementary material for: Diversity and functional structure of soil animal communities suggest soil animal food webs to be buffered against changes in forest land use
Source: Oecologia. 2021 Apr 14;196(1):195–209. doi: 10.1007/s00442-021-04910-1 (PMC8139884; doi:10.1007/s00442-021-04910-1)
Supplement: Supplementary file 2 — Supplementary file2 (DOCX 1559 kb) [file 442_2021_4910_MOESM2_ESM.docx]

**Appendix S2**
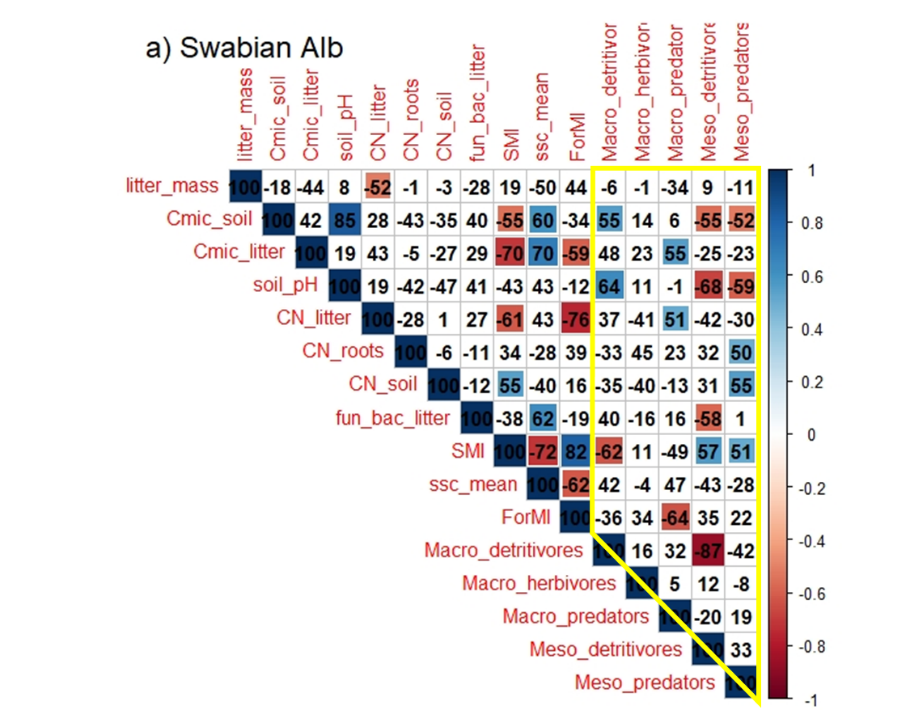

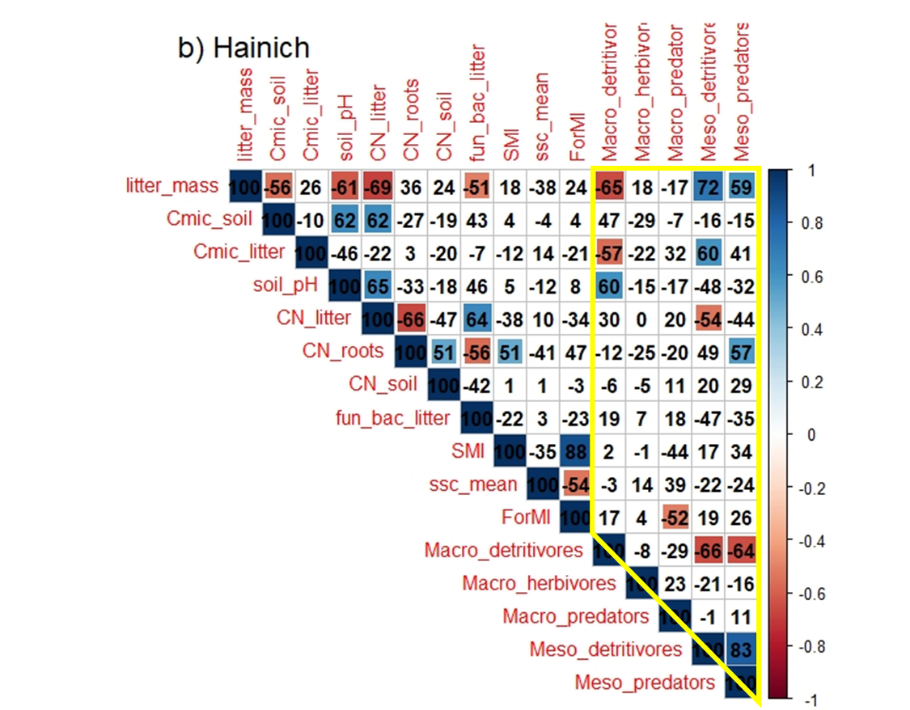

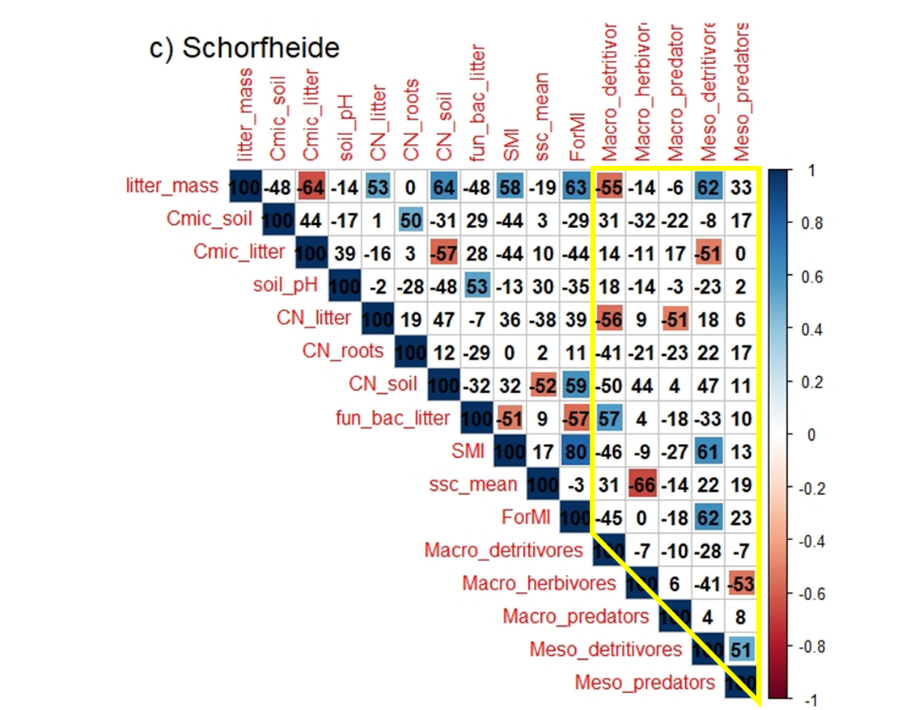


**Fig. S1:** Correlation plots for the regions a) Swabian Alb, b) Hainich and c) Schorfheide showing linear correlation coefficients between the biomass of macrofauna herbivores, detritivores and predators (Macro_herbivores, Macro_detritivores and Macro_predators, respectively), the biomass of mesofauna detritivores and predators (Meso_detritivores and Meso_predators, respectively), environmental factors [C-to-N ratios in leaf litter, roots and soil, amount of leaf litter – litter_mass, microbial biomass in leaf litter and soil – Cmic_litter and Cmic_soil, and different indices describing forest management (ForMI – Forest Management Intensity, SMI - silvicultural management intensity and ssc_mean – stand structural complexity)]. Coefficients were converted to percentages for spacesaving. Significant correlations (p ≤ 0.05) are shown with colored squares; the size of the squares represents the strength of the correlation; small < 0.40, medium 0.41 – 0.60, large > 0.61.

Correlations of soil animal groups with environmental factors are framed in yellow.
