# Supplementary material for: Diversity and functional structure of soil animal communities suggest soil animal food webs to be buffered against changes in forest land use
Source: Oecologia. 2021 Apr 14;196(1):195–209. doi: 10.1007/s00442-021-04910-1 (PMC8139884; doi:10.1007/s00442-021-04910-1)
Supplement: Supplementary file 3 — Supplementary file3 (DOCX 28 kb) [file 442_2021_4910_MOESM3_ESM.docx]

**Appendix S4**

**Table S1:** Mean (± SD) values for the measured environmental variables, including the amount of leaf litter, the microbial biomass of leaf litter and soil (soil Cmic and leaf litter Cmic, respectively), the soil pH and the C-to-N ratios of soil, leaf litter and fine roots. Means are reported for regions (Swabian Alb – SA, Hainich – Hai and Schorfheide-Chorin – Sch), forest types (coniferous – Conif, young beech – B30, old beech – B70 and natural beech – Bnat) and for forest types within regions.

| Factorlevel | N | Amount of leaf litter (g/cm²) | Soil Cmic (µg/g) | Leaf litter Cmic (µg/g) | Soil pH | Leaf litter C/N | Fine root C/N | Soil C/N |
| --- | --- | --- | --- | --- | --- | --- | --- | --- |
| Total | 48 | 0.57 ± 0.31 | 824.98 ± 511.24 | 7461.46 ± 3055.06 | 4.15 ± 0.82 | 32.61 ± 5.51 | 33.56 ± 4.81 | 15.82 ± 2.93 |
| SA | 16 | 0.72 ± 0.26 | 1109.53 ± 468.41 | 8568.89 ± 2864.33 | 4.53 ± 0.71 | 31.69 ± 3.52 | 35.11 ± 5.57 | 14.41 ± 1.98 |
| Hai | 16 | 0.45 ± 0.30 | 1016.02 ± 473.09 | 6330.73 ± 2824.69 | 4.61 ± 0.67 | 31.23 ± 3.97 | 32.06 ± 4.18 | 14.85 ± 2.35 |
| Sch | 16 | 0.56 ± 0.32 | 349.40 ± 95.76 | 7484.76 ± 3223.16 | 3.32 ± 0.20 | 34.91 ± 7.65 | 33.51 ± 4.34 | 18.20 ± 2.86 |
| Conif | 12 | 0.79 ± 0.37 | 599.03 ± 437.60 | 5105.13 ± 2680.39 | 3.78 ± 0.84 | 33.44 ± 9.50 | 36.53 ± 5.56 | 17.93 ± 3.46 |
| B30 | 12 | 0.62 ± 0.33 | 934.04 ± 504.53 | 7627.39 ± 2753.16 | 4.45 ± 0.82 | 30.89 ± 3.53 | 33.17 ± 3.62 | 15.31 ± 2.93 |
| B70 | 12 | 0.45 ± 0.17 | 850.86 ± 501.72 | 7349.19 ± 2636.65 | 4.28 ± 0.88 | 32.32 ± 2.80 | 32.50 ± 3.21 | 14.36 ± 2.12 |
| Bnat | 12 | 0.44 ± 0.22 | 916.00 ± 583.55 | 9764.12 ± 2505.50 | 4.10 ± 0.66 | 33.78 ± 3.70 | 32.03 ± 5.56 | 15.69 ± 2.00 |
| SA, Conif | 4 | 0.79 ± 0.34 | 613.70 ± 245.09 | 5895.74 ± 2321.71 | 3.75 ± 0.54 | 29.03 ± 1.70 | 38.38 ± 7.97 | 16.35 ± 1.53 |
| SA, B30 | 4 | 0.92 ± 0.22 | 1161.91 ± 109.49 | 7819.44 ± 1185.05 | 5.00 ± 0.35 | 30.08 ± 3.25 | 35.13 ± 4.10 | 13.95 ± 2.44 |
| SA, B70 | 4 | 0.63 ± 0.03 | 1258.42 ± 604.22 | 8129.34 ± 1762.11 | 4.75 ± 0.77 | 33.05 ± 3.27 | 33.48 ± 4.38 | 12.93 ± 1.45 |
| SA, Bnat | 4 | 0.52 ± 0.23 | 1404.08 ± 428.02 | 12431.04 ± 836.54 | 4.60 ± 0.55 | 34.60 ± 3.32 | 33.45 ± 5.87 | 14.43 ± 1.02 |
| Hai, Conif | 4 | 0.65 ± 0.46 | 864.97 ± 655.67 | 4813.02 ± 3428.65 | 4.43 ± 1.08 | 27.53 ± 5.61 | 35.63 ± 3.53 | 15.53 ± 2.50 |
| Hai, B30 | 4 | 0.31 ± 0.18 | 1346.15 ± 274.69 | 7249.71 ± 3927.80 | 4.93 ± 0.43 | 32.03 ± 3.39 | 32.68 ± 3.83 | 15.15 ± 3.44 |
| Hai, B70 | 4 | 0.38 ± 0.14 | 899.84 ± 228.86 | 5939.19 ± 2375.13 | 4.78 ± 0.55 | 33.40 ± 2.54 | 31.83 ± 3.25 | 13.83 ± 1.73 |
| Hai, Bnat | 4 | 0.47 ± 0.29 | 953.13 ± 601.94 | 7321.02 ± 1166.51 | 4.30 ± 0.47 | 31.98 ± 1.78 | 28.10 ± 3.38 | 14.90 ± 2.09 |
| Sch, Conif | 4 | 0.93 ± 0.34 | 318.44 ± 112.30 | 4606.64 ± 2814.00 | 3.15 ± 0.24 | 43.78 ± 9.04 | 35.60 ± 5.52 | 21.90 ± 1.83 |
| Sch, B30 | 4 | 0.62 ± 0.26 | 294.06 ± 64.32 | 7813.03 ± 3267.38 | 3.43 ± 0.17 | 30.58 ± 4.59 | 31.70 ± 2.86 | 16.83 ± 2.86 |
| Sch, B70 | 4 | 0.34 ± 0.11 | 394.32 ± 54.38 | 7979.06 ± 3571.10 | 3.30 ± 0.14 | 30.50 ± 2.20 | 32.20 ± 2.46 | 16.33 ± 1.76 |
| Sch, Bnat | 4 | 0.35 ± 0.14 | 390.79 ± 125.72 | 9540.31 ± 1857.06 | 3.40 ± 0.16 | 34.78 ± 5.44 | 34.55 ± 5.96 | 17.75 ± 0.79 |

**Table S2:** Number of individuals per m² (mean ± SD) reported for regions (Swabian Alb – SA, Hainich – Hai and Schorfheide-Chorin – Sch), forest types (coniferous – Conif, young beech – B30, old beech – B70 and natural beech – Bnat) and for forest types within regions.

| Factorlevel | N | Macrofauna decomposers | Macrofauna herbivores | Macrofauna predators | Mesofauna decomposers | Mesofauna predators |
| --- | --- | --- | --- | --- | --- | --- |
| Total | 48 | 690.67 ± 606.34 | 156.52 ± 123.40 | 867.06 ± 377.14 | 87234.38 ± 57740.73 | 40742.31 ± 33548.46 |
| Alb | 16 | 877.75 ± 661.07 | 147.75 ± 98.95 | 856.50 ± 418.73 | 76232.13 ± 44959.06 | 41924.69 ± 24823.13 |
| Hai | 16 | 970.44 ± 507.17 | 183.31 ± 158.27 | 938.19 ± 384.13 | 71907.94 ± 64163.84 | 33284.06 ± 36739.91 |
| Sch | 16 | 223.81 ± 323.14 | 138.50 ± 108.06 | 806.50 ± 336.68 | 113563.06 ± 56472.12 | 47018.19 ± 38180.06 |
| Conif | 12 | 594.17 ± 494.72 | 204.58 ± 169.75 | 793.08 ± 385.07 | 145731.58 ± 67337.42 | 56774.08 ± 41926.77 |
| B30 | 12 | 549.42 ± 340.38 | 100.42 ± 89.59 | 762.75 ± 303.08 | 79540.17 ± 45865.67 | 41369.75 ± 20283.24 |
| B70 | 12 | 849.08 ± 892.40 | 135.50 ± 91.72 | 855.50 ± 425.89 | 65753.17 ± 43811.42 | 37937.50 ± 43993.18 |
| unm B | 12 | 770.00 ± 589.67 | 185.58 ± 111.58 | 1056.92 ± 359.03 | 57912.58 ± 21187.25 | 26887.92 ± 14335.18 |
| Alb, Conif | 4 | 489.50 ± 250.39 | 168.00 ± 94.73 | 840.50 ± 430.17 | 136359.00 ± 42695.28 | 66069.00 ± 27871.30 |
| Alb, B30 | 4 | 747.00 ± 426.23 | 93.25 ± 87.54 | 548.75 ± 347.78 | 51099.75 ± 31937.38 | 37336.00 ± 22332.68 |
| Alb, B70 | 4 | 1175.75 ± 1087.91 | 109.75 ± 73.92 | 714.00 ± 301.50 | 47408.00 ± 17138.64 | 25591.25 ± 22468.82 |
| Alb, unm B | 4 | 1098.75 ± 591.51 | 220.00 ± 116.04 | 1322.75 ± 190.31 | 70061.75 ± 12203.51 | 38702.50 ± 11209.11 |
| Hai, Conif | 4 | 1093.25 ± 501.65 | 246.00 ± 245.74 | 815.00 ± 254.10 | 132729.25 ± 103829.77 | 54597.50 ± 70367.25 |
| Hai, B30 | 4 | 587.50 ± 111.42 | 142.00 ± 122.25 | 852.25 ± 304.53 | 67578.00 ± 44857.38 | 34685.00 ± 19173.75 |
| Hai, B70 | 4 | 1316.00 ± 678.39 | 169.50 ± 113.76 | 1151.50 ± 555.56 | 45626.75 ± 11242.61 | 27876.00 ± 15551.62 |
| Hai, unm B | 4 | 885.00 ± 421.02 | 175.75 ± 170.68 | 934.00 ± 425.50 | 41697.75 ± 23888.74 | 15977.75 ± 11892.28 |
| Sch, Conif | 4 | 199.75 ± 170.07 | 199.75 ± 179.41 | 723.75 ± 532.93 | 168106.50 ± 54884.48 | 49655.75 ± 22978.99 |
| Sch, B30 | 4 | 313.75 ± 320.46 | 66.00 ± 53.59 | 887.25 ± 175.27 | 119942.75 ± 35149.35 | 52088.25 ± 20190.70 |
| Sch, B70 | 4 | 55.50 ± 60.42 | 127.25 ± 99.63 | 701.00 ± 300.27 | 104224.75 ± 60461.39 | 60345.25 ± 73089.48 |
| Sch, unm B | 4 | 326.25 ± 568.79 | 161.00 ± 23.27 | 914.00 ± 337.32 | 61978.25 ± 18893.48 | 25983.50 ± 11853.58 |

**Table S3:** Number of species (mean ± SD) reported for regions (Swabian Alb – SA, Hainich – Hai and Schorfheide-Chorin – Sch), forest types (coniferous – Conif, young beech – B30, old beech – B70 and natural beech – Bnat) and for forest types within regions.

| Factorlevel | N | Macrofauna decomposers | Macrofauna herbivores | Macrofauna predators | Mesofauna decomposers | Mesofauna predators |
| --- | --- | --- | --- | --- | --- | --- |
| Total | 48 | 9.60 ± 5.61 | 2.83 ± 1.59 | 18.75 ± 5.06 | 23.65 ± 4.81 | 16.60 ± 4.29 |
| Alb | 16 | 10.75 ± 4.14 | 3.06 ± 1.77 | 18.88 ± 4.00 | 24.19 ± 5.04 | 16.81 ± 3.58 |
| Hai | 16 | 14.19 ± 3.64 | 3.00 ± 1.41 | 21.31 ± 4.74 | 22.44 ± 4.24 | 17.81 ± 5.75 |
| Sch | 16 | 3.88 ± 3.10 | 2.44 ± 1.59 | 16.06 ± 5.18 | 24.31 ± 5.19 | 15.19 ± 2.81 |
| Conif | 12 | 7.67 ± 6.44 | 2.67 ± 1.30 | 17.92 ± 4.14 | 26.67 ± 5.61 | 16.58 ± 2.61 |
| B30 | 12 | 11.08 ± 4.60 | 2.83 ± 1.75 | 19.17 ± 5.24 | 23.67 ± 4.83 | 18.58 ± 4.21 |
| B70 | 12 | 10.00 ± 6.37 | 1.92 ± 1.08 | 17.00 ± 6.22 | 22.67 ± 3.58 | 16.00 ± 5.49 |
| unm B | 12 | 9.67 ± 4.98 | 3.92 ± 1.62 | 20.92 ± 4.06 | 21.58 ± 3.96 | 15.25 ± 4.14 |
| Alb, Conif | 4 | 5.75 ± 2.87 | 2.50 ± 1.29 | 20.00 ± 2.94 | 31.25 ± 3.77 | 18.50 ± 3.51 |
| Alb, B30 | 4 | 12.75 ± 1.50 | 3.00 ± 1.83 | 18.50 ± 5.74 | 21.00 ± 1.41 | 17.50 ± 3.42 |
| Alb, B70 | 4 | 12.00 ± 5.35 | 1.75 ± 1.26 | 16.00 ± 2.58 | 21.50 ± 4.12 | 14.00 ± 4.24 |
| Alb, unm B | 4 | 12.50 ± 1.29 | 5.00 ± 1.15 | 21.00 ± 3.56 | 23.00 ± 1.41 | 17.25 ± 2.63 |
| Hai, Conif | 4 | 15.50 ± 3.11 | 3.25 ± 0.96 | 17.75 ± 1.71 | 20.75 ± 3.40 | 15.25 ± 1.71 |
| Hai, B30 | 4 | 14.25 ± 3.77 | 3.75 ± 1.71 | 22.50 ± 5.74 | 24.25 ± 5.74 | 21.00 ± 5.60 |
| Hai, B70 | 4 | 14.75 ± 3.95 | 1.75 ± 0.96 | 23.00 ± 5.83 | 22.25 ± 1.50 | 20.75 ± 6.75 |
| Hai, unm B | 4 | 12.25 ± 4.35 | 3.25 ± 1.50 | 22.00 ± 4.40 | 22.50 ± 5.92 | 14.25 ± 5.91 |
| Sch, Conif | 4 | 1.75 ± 0.96 | 2.25 ± 1.71 | 16.00 ± 6.38 | 28.00 ± 3.56 | 16.00 ± 1.41 |
| Sch, B30 | 4 | 6.25 ± 3.59 | 1.75 ± 1.50 | 16.50 ± 3.11 | 25.75 ± 5.91 | 17.25 ± 3.20 |
| Sch, B70 | 4 | 3.25 ± 2.87 | 2.25 ± 1.26 | 12.00 ± 4.32 | 24.25 ± 4.72 | 13.25 ± 1.26 |
| Sch, unm B | 4 | 4.25 ± 3.40 | 3.50 ± 1.91 | 19.75 ± 4.99 | 19.25 ± 3.10 | 14.25 ± 3.59 |

**Table S4:** Biomass (g/m², mean ± SD) of soil animal functional groups. Means and number of replicates (N) are reported for regions (Swabian Alb – SA, Hainich – Hai and Schorfheide-Chorin – Sch), forest types (coniferous – Conif, young beech – B30, old beech – B70 and natural beech – Bnat) and for forest types within regions.

| Factorlevel | N | Macrofauna decomposers | Macrofauna herbivores | Macrofauna predators | Mesofauna decomposers | Mesofauna predators |
| --- | --- | --- | --- | --- | --- | --- |
| Total | 48 | 23677.54 ± 26214.08 | 609.11 ± 608.5 | 4520.28 ± 3981.81 | 1269.03 ± 802.96 | 566.92 ± 396.03 |
| Alb | 16 | 27087.21 ± 23991.06 | 560.07 ± 576.71 | 3699.52 ± 2585.93 | 1163.47 ± 704.29 | 691.19 ± 535.06 |
| Hai | 16 | 40590.26 ± 28364.88 | 770.21 ± 546.94 | 5107.91 ± 4298.28 | 916.68 ± 457.39 | 465.22 ± 362.33 |
| Sch | 16 | 3355.16 ± 3870.23 | 497.05 ± 696.27 | 4753.41 ± 4834.40 | 1726.94 ± 973.15 | 544.36 ± 214.02 |
| Conif | 12 | 23015.95 ± 37896.05 | 904.03 ± 985.44 | 2450.50 ± 1255.15 | 1989.64 ± 1136.48 | 721.76 ± 450.06 |
| B30 | 12 | 19703.45 ± 16310.67 | 404.63 ± 396.30 | 4287.04 ± 2586.53 | 1161.94 ± 612.09 | 649.09 ± 548.69 |
| B70 | 12 | 24696.44 ± 20260.94 | 449.01 ± 296.06 | 4661.96 ± 3216.57 | 1024.35 ± 469.54 | 466.76 ± 267.23 |
| unm B | 12 | 27294.34 ± 28113.36 | 678.79 ± 438.75 | 6681.61 ± 6267.44 | 900.21 ± 274.68 | 430.08 ± 173.82 |
| Alb, Conif | 4 | 5164.56 ± 5960.56 | 879.10 ± 1000.41 | 2387.73 ± 1114.42 | 2141.78 ± 463.59 | 994.04 ± 357.78 |
| Alb, B30 | 4 | 30070.09 ± 17551.96 | 432.48 ± 454.43 | 2676.97 ± 1254.89 | 782.80 ± 538.25 | 868.22 ± 948.19 |
| Alb, B70 | 4 | 32457.53 ± 29258.30 | 410.61 ± 368.18 | 3766.55 ± 1753.32 | 821.60 ± 439.94 | 388.73 ± 255.97 |
| Alb, unm B | 4 | 40656.66 ± 27231.96 | 518.10 ± 361.32 | 5966.84 ± 4122.55 | 907.71 ± 263.74 | 513.77 ± 104.13 |
| Hai, Conif | 4 | 63712.71 ± 43601.87 | 890.05 ± 829.84 | 1857.90 ± 200.54 | 1048.95 ± 744.63 | 598.05 ± 648.04 |
| Hai, B30 | 4 | 26853.75 ± 6530.56 | 601.79 ± 481.53 | 5007.00 ± 2695.47 | 967.87 ± 447.21 | 501.14 ± 269.29 |
| Hai, B70 | 4 | 33034.34 ± 10899.90 | 556.71 ± 265.24 | 6880.89 ± 4346.24 | 868.46 ± 384.41 | 433.49 ± 241.56 |
| Hai, unm B | 4 | 38760.24 ± 30252.49 | 1032.30 ± 543.60 | 6685.83 ± 6676.26 | 781.45 ± 299.14 | 328.20 ± 232.3 |
| Sch, Conif | 4 | 170.57 ± 94.97 | 942.93 ± 1366.78 | 3105.87 ± 1856.91 | 2778.18 ± 1388.03 | 573.18 ± 214.57 |
| Sch, B30 | 4 | 2186.52 ± 2042.11 | 179.62 ± 130.81 | 5177.16 ± 3238.24 | 1735.15 ± 451.68 | 577.91 ± 179.85 |
| Sch, B70 | 4 | 8597.45 ± 3428.56 | 379.69 ± 302.73 | 3338.44 ± 2450.11 | 1382.99 ± 456.46 | 578.07 ± 334.44 |
| Sch, unm B | 4 | 2466.13 ± 2433.14 | 485.97 ± 170.75 | 7392.17 ± 9005.96 | 1011.45 ± 286.76 | 448.27 ± 149.51 |
