# Supplementary material for: Diversity and functional structure of soil animal communities suggest soil animal food webs to be buffered against changes in forest land use
Source: Oecologia. 2021 Apr 14;196(1):195–209. doi: 10.1007/s00442-021-04910-1 (PMC8139884; doi:10.1007/s00442-021-04910-1)
Supplement: Supplementary file 4 — Supplementary file4 (DOCX 76 kb) [file 442_2021_4910_MOESM4_ESM.docx]

**Appendix 1**

**Table S1:** Complete list of species found across all regions and forest types, including the taxonomic group, taxonomic authority, family and functional group.

| **Taxonomic group** | **Species** | **Authority** | **Family** | **Functional group** |
| --- | --- | --- | --- | --- |
| Araneae | *Agroeca brunnea* | (Blackwall, 1833) | Liocranidae | Macrofauna predators |
| Araneae | *Amaurobius fenestralis* | (Ström, 1768) | Amaurobiidae | Macrofauna predators |
| Araneae | *Anyphaena accentuata* | (Walckenaer, 1802) | Anyphaenidae | Macrofauna predators |
| Araneae | *Apostenus fuscus* | Westring, 1851 | Liocranidae | Macrofauna predators |
| Araneae | *Araniella cucurbitina* | (Clerck, 1757) | Araneidae | Macrofauna predators |
| Araneae | *Asthenargus paganus* | (Simon, 1884) | Linyphiidae | Macrofauna predators |
| Araneae | *Ballus chalybeius* | (Walckenaer, 1802) | Salticidae | Macrofauna predators |
| Araneae | *Bathyphantes gracilis* | (Blackwall, 1841) | Linyphiidae | Macrofauna predators |
| Araneae | *Brigittea latens* | (Fabricius, 1775) | Dictynidae | Macrofauna predators |
| Araneae | *Callobius claustrarius* | (Hahn, 1833) | Amaurobiidae | Macrofauna predators |
| Araneae | *Centromerus brevipalpus* | (Menge, 1866) | Linyphiidae | Macrofauna predators |
| Araneae | *Centromerus cavernarum* | (L. Koch, 1872) | Linyphiidae | Macrofauna predators |
| Araneae | *Centromerus prudens* | (O. Pickard-Cambridge, 1873) | Linyphiidae | Macrofauna predators |
| Araneae | *Centromerus serratus* | (O. Pickard-Cambridge, 1875) | Linyphiidae | Macrofauna predators |
| Araneae | *Centromerus sylvaticus* | (Blackwall, 1841) | Linyphiidae | Macrofauna predators |
| Araneae | *Ceratinella brevis* | (Wider, 1834) | Linyphiidae | Macrofauna predators |
| Araneae | *Ceratinella scabrosa* | (O. Pickard-Cambridge, 1871) | Linyphiidae | Macrofauna predators |
| Araneae | *Clubiona comta* | C. L. Koch, 1839 | Clubionidae | Macrofauna predators |
| Araneae | *Clubiona pallidula* | (Clerck, 1757) | Clubionidae | Macrofauna predators |
| Araneae | *Coelotes terrestris* | (Wider, 1834) | Agelenidae | Macrofauna predators |
| Araneae | *Dicymbium nigrum* | (Blackwall, 1834) | Linyphiidae | Macrofauna predators |
| Araneae | *Diplocephalus latifrons* | (O. Pickard-Cambridge, 1863) | Linyphiidae | Macrofauna predators |
| Araneae | *Diplocephalus picinus* | (Blackwall, 1841) | Linyphiidae | Macrofauna predators |
| Araneae | *Diplostyla concolor* | (Wider, 1834) | Linyphiidae | Macrofauna predators |
| Araneae | *Donacochara speciosa* | (Thorell, 1875) | Linyphiidae | Macrofauna predators |
| Araneae | *Enoplognatha ovata* | (Clerck, 1757) | Theridiidae | Macrofauna predators |
| Araneae | *Erigonella hiemalis* | (Blackwall, 1841) | Linyphiidae | Macrofauna predators |
| Araneae | *Ero furcata* | (Villers, 1789) | Mimetidae | Macrofauna predators |
| Araneae | *Euophrys frontalis* | (Walckenaer, 1802) | Salticidae | Macrofauna predators |
| Araneae | *Euophrys herbigrada* | (Simon, 1871) | Salticidae | Macrofauna predators |
| Araneae | *Euryopis flavomaculata* | (C. L. Koch, 1836) | Theridiidae | Macrofauna predators |
| Araneae | *Evarcha arcuata* | (Clerck, 1757) | Salticidae | Macrofauna predators |
| Araneae | *Gonatium rubens* | (Blackwall, 1833) | Linyphiidae | Macrofauna predators |
| Araneae | *Gongylidiellum latebricola* | (O. Pickard-Cambridge, 1871) | Linyphiidae | Macrofauna predators |
| Araneae | *Hahnia pusilla* | C. L. Koch, 1841 | Hahniidae | Macrofauna predators |
| Araneae | *Haplodrassus silvestris* | (Blackwall, 1833) | Gnaphosidae | Macrofauna predators |
| Araneae | *Haplodrassus soerenseni* | (Strand, 1900) | Gnaphosidae | Macrofauna predators |
| Araneae | *Harpactea lepida* | (C. L. Koch, 1838) | Dysderidae | Macrofauna predators |
| Araneae | *Histopona torpida* | (C. L. Koch, 1837) | Agelenidae | Macrofauna predators |
| Araneae | *Inermocoelotes inermis* | (L. Koch, 1855) | Agelenidae | Macrofauna predators |
| Araneae | *Linyphia hortensis* | Sundevall, 1830 | Linyphiidae | Macrofauna predators |
| Araneae | *Macrargus rufus* | (Wider, 1834) | Linyphiidae | Macrofauna predators |
| Araneae | *Maro minutus* | O. Pickard-Cambridge, 1906 | Linyphiidae | Macrofauna predators |
| Araneae | *Metellina segmentata* | (Clerck, 1757) | Tetragnathidae | Macrofauna predators |
| Araneae | *Micrargus herbigradus* | (Blackwall, 1854) | Linyphiidae | Macrofauna predators |
| Araneae | *Microlinyphia pusilla* | (Sundevall, 1830) | Linyphiidae | Macrofauna predators |
| Araneae | *Microneta viaria* | (Blackwall, 1841) | Linyphiidae | Macrofauna predators |
| Araneae | *Monocephalus fuscipes* | (Blackwall, 1836) | Linyphiidae | Macrofauna predators |
| Araneae | *Neon reticulatus* | (Blackwall, 1853) | Salticidae | Macrofauna predators |
| Araneae | *Nusoncus nasutus* | Wunderlich, 2008 | Linyphiidae | Macrofauna predators |
| Araneae | *Ozyptila praticola* | (C. L. Koch, 1837) | Thomisidae | Macrofauna predators |
| Araneae | *Ozyptila trux* | (Blackwall, 1846) | Thomisidae | Macrofauna predators |
| Araneae | *Pachygnatha degeeri* | Sundevall, 1830 | Tetragnathidae | Macrofauna predators |
| Araneae | *Palliduphantes pallidus* | (O. Pickard-Cambridge, 1871) | Linyphiidae | Macrofauna predators |
| Araneae | *Pardosa lugubris* | (Walckenaer, 1802) | Lycosidae | Macrofauna predators |
| Araneae | *Pelecopsis radicicola* | (L. Koch, 1872) | Linyphiidae | Macrofauna predators |
| Araneae | *Philodromus dispar* | Walckenaer, 1826 | Philodromidae | Macrofauna predators |
| Araneae | *Pocadicnemis juncea* | Locket & Millidge, 1953 | Linyphiidae | Macrofauna predators |
| Araneae | *Porrhomma microphthalmum* | (O. Pickard-Cambridge, 1871) | Linyphiidae | Macrofauna predators |
| Araneae | *Robertus lividus* | (Blackwall, 1836) | Theridiidae | Macrofauna predators |
| Araneae | *Robertus scoticus* | Jackson, 1914 | Theridiidae | Macrofauna predators |
| Araneae | *Saaristoa abnormis* | (Blackwall, 1841) | Linyphiidae | Macrofauna predators |
| Araneae | *Saloca diceros* | (O. Pickard-Cambridge, 1871) | Linyphiidae | Macrofauna predators |
| Araneae | *Tapinocyba insecta* | (L. Koch, 1869) | Linyphiidae | Macrofauna predators |
| Araneae | *Tapinocyba pallens* | (O. Pickard-Cambridge, 1872) | Linyphiidae | Macrofauna predators |
| Araneae | *Tapinocyba praecox* | (O. Pickard-Cambridge, 1873) | Linyphiidae | Macrofauna predators |
| Araneae | *Tenuiphantes flavipes* | (Blackwall, 1854) | Linyphiidae | Macrofauna predators |
| Araneae | *Tenuiphantes mengei* | (Kulczyński, 1887) | Linyphiidae | Macrofauna predators |
| Araneae | *Tenuiphantes tenebricola* | (Wider, 1834) | Linyphiidae | Macrofauna predators |
| Araneae | *Tenuiphantes tenuis* | (Blackwall, 1852) | Linyphiidae | Macrofauna predators |
| Araneae | *Walckenaeria antica* | (Wider, 1834) | Linyphiidae | Macrofauna predators |
| Araneae | *Walckenaeria atrotibialis* | (O. Pickard-Cambridge, 1878) | Linyphiidae | Macrofauna predators |
| Araneae | *Walckenaeria corniculans* | (O. Pickard-Cambridge, 1875) | Linyphiidae | Macrofauna predators |
| Araneae | *Walckenaeria cucullata* | (C. L. Koch, 1836) | Linyphiidae | Macrofauna predators |
| Araneae | *Walckenaeria cuspidata* | Blackwall, 1833 | Linyphiidae | Macrofauna predators |
| Araneae | *Walckenaeria dysderoides* | (Wider, 1834) | Linyphiidae | Macrofauna predators |
| Araneae | *Walckenaeria furcillata* | (Menge, 1869) | Linyphiidae | Macrofauna predators |
| Araneae | *Walckenaeria nudipalpis* | (Westring, 1851) | Linyphiidae | Macrofauna predators |
| Araneae | *Xysticus cristatus* | (Clerck, 1757) | Thomisidae | Macrofauna predators |
| Araneae | *Xysticus erraticus* | (Blackwall, 1834) | Thomisidae | Macrofauna predators |
| Araneae | *Xysticus lanio* | C. L. Koch, 1835 | Thomisidae | Macrofauna predators |
| Araneae | *Zora spinimana* | (Sundevall, 1833) | Miturgidae | Macrofauna predators |
| Chilopoda | *Cryptops hortensis* | Leach, 1815 | Cryptopidae | Macrofauna predators |
| Chilopoda | *Geophilus electricus* | Linnaeus, 1758 | Geophilidae | Macrofauna predators |
| Chilopoda | *Geophilus flavus* | De Geer, 1778 | Geophilidae | Macrofauna predators |
| Chilopoda | *Geophilus insculptus* | Attems, 1895 | Geophilidae | Macrofauna predators |
| Chilopoda | *Geophilus ribauti* | Brölemann, 1908 | Geophilidae | Macrofauna predators |
| Chilopoda | *Geophilus studeri* | Rothenbühler, 1899 | Geophilidae | Macrofauna predators |
| Chilopoda | *Geophilus truncorum* | Bergsøe & Meinert, 1866 | Geophilidae | Macrofauna predators |
| Chilopoda | *Lithobius aeruginosus* | L. Koch, 1862 | Lithobiidae | Macrofauna predators |
| Chilopoda | *Lithobius calcaratus* | C. L. Koch, 1844 | Lithobiidae | Macrofauna predators |
| Chilopoda | *Lithobius crassipes* | L. Koch, 1862 | Lithobiidae | Macrofauna predators |
| Chilopoda | *Lithobius curtipes* | C. L. Koch, 1847 | Lithobiidae | Macrofauna predators |
| Chilopoda | *Lithobius dentatus* | C. L. Koch, 1844 | Lithobiidae | Macrofauna predators |
| Chilopoda | *Lithobius erythrocephalus* | C. L. Koch, 1847 | Lithobiidae | Macrofauna predators |
| Chilopoda | *Lithobius forficatus* | Linnaeus, 1758 | Lithobiidae | Macrofauna predators |
| Chilopoda | *Lithobius lapidicola* | Meinert, 1872 | Lithobiidae | Macrofauna predators |
| Chilopoda | *Lithobius macilentus* | L. Koch, 1862 | Lithobiidae | Macrofauna predators |
| Chilopoda | *Lithobius melanops* | Newport, 1845 | Lithobiidae | Macrofauna predators |
| Chilopoda | *Lithobius mutabilis* | L. Koch, 1862 | Lithobiidae | Macrofauna predators |
| Chilopoda | *Lithobius muticus* | C. L. Koch, 1847 | Lithobiidae | Macrofauna predators |
| Chilopoda | *Lithobius piceus* | L. Koch, 1862 | Lithobiidae | Macrofauna predators |
| Chilopoda | *Lithobius subtilis* | Latzel, 1880 | Lithobiidae | Macrofauna predators |
| Chilopoda | *Lithobius tricuspis* | Meinert, 1872 | Lithobiidae | Macrofauna predators |
| Chilopoda | *Schendyla nemorensis* | C. L.Koch, 1837 | Schendylidae | Macrofauna predators |
| Chilopoda | *Strigamia acuminata* | Leach, 1815 | Linotaeniidae | Macrofauna predators |
| Coleoptera | *Abax ovalis* | (Duftschmid, 1812) | Carabidae | Macrofauna predators |
| Coleoptera | *Abax parallelepipedus* | (Piller & Mitterpacher, 1783) | Carabidae | Macrofauna predators |
| Coleoptera | *Abax parallelus* | (Duftschmid, 1812) | Carabidae | Macrofauna predators |
| Coleoptera | *Acrotona sylvicola* | (Kraatz, 1856) | Staphylinidae | Macrofauna predators |
| Coleoptera | *Agonum sexpunctatum* | (Linnaeus, 1758) | Carabidae | Macrofauna predators |
| Coleoptera | *Agriotes acuminatus* | (Stephens, 1830) | Elateridae | Macrofauna herbivores |
| Coleoptera | *Agriotinae* sp.1 |  | Elateridae | Macrofauna herbivores |
| Coleoptera | *Aleocharinae* sp.1 |  | Staphylinidae | Macrofauna predators |
| Coleoptera | *Aleocharinae* sp.2 |  | Staphylinidae | Macrofauna predators |
| Coleoptera | *Amara aenea* | (De Geer, 1774) | Carabidae | Macrofauna predators |
| Coleoptera | *Anatis ocellata* | (Linnaeus, 1758) | Coccinellidae | Macrofauna predators |
| Coleoptera | *Anthicus flavipes* | (Panzer, 1797) | Anthicidae | Macrofauna decomposers |
| Coleoptera | *Atheta lativentris* | J.Sahlberg, 1876 | Staphylinidae | Macrofauna predators |
| Coleoptera | *Athous haemorrhoidalis* | (Fabricius, 1801) | Elateridae | Macrofauna herbivores |
| Coleoptera | *Athous mollis* | Reitter, 1889 | Elateridae | Macrofauna herbivores |
| Coleoptera | *Athous subfuscus* | (Müller, 1764) | Elateridae | Macrofauna herbivores |
| Coleoptera | *Athous vittatus* | (Fabricius, 1792) | Elateridae | Macrofauna herbivores |
| Coleoptera | *Athous zebei* | Bach, 1852 | Elateridae | Macrofauna herbivores |
| Coleoptera | *Byrrhidae* sp.1 |  | Byrrhidae | Macrofauna herbivores |
| Coleoptera | *Bythinus acutangulus* | Reitter, 1878 | Staphylinidae | Macrofauna predators |
| Coleoptera | *Calathus melanocephalus* | (Linnaeus, 1758) | Carabidae | Macrofauna predators |
| Coleoptera | *Carabus nemoralis* | O. F. Müller, 1764 | Carabidae | Macrofauna predators |
| Coleoptera | *Coccinella septempunctata* | Linnaeus, 1758 | Coccinellidae | Macrofauna predators |
| Coleoptera | *Cryptorhynchinae* sp.1 |  | Curculionidae | Macrofauna herbivores |
| Coleoptera | *Cychrus attenuatus* | (Fabricius, 1792) | Carabidae | Macrofauna predators |
| Coleoptera | *Cychrus caraboides* | (Linnaeus, 1758) | Carabidae | Macrofauna predators |
| Coleoptera | *Dalopius marginatus* | (Linnaeus, 1758) | Elateridae | Macrofauna herbivores |
| Coleoptera | *Domene scabricollis* | (Erichson, 1840) | Staphylinidae | Macrofauna predators |
| Coleoptera | *Ectinus aterrimus* | (Linnaeus, 1760) | Elateridae | Macrofauna herbivores |
| Coleoptera | *Elater ferrugineus* | Linnaeus, 1758 | Elateridae | Macrofauna herbivores |
| Coleoptera | *Geostiba circellaris* | (Gravenhorst, 1806) | Staphylinidae | Macrofauna predators |
| Coleoptera | *Habrocerus capillaricornis* | (Gravenhorst, 1806) | Staphylinidae | Macrofauna predators |
| Coleoptera | *Harpalus affinis* | (Schrank, 1781) | Carabidae | Macrofauna predators |
| Coleoptera | *Harpalus latus* | (Panzer, 1796) | Carabidae | Macrofauna predators |
| Coleoptera | *Heterothops dissimilis* | (Gravenhorst, 1802) | Staphylinidae | Macrofauna predators |
| Coleoptera | *Histeridae* sp.1 |  | Histeridae | Macrofauna predators |
| Coleoptera | *Lamprohiza splendidula* | (Linnaeus, 1767) | Lampyridae | Macrofauna predators |
| Coleoptera | *Lathrobium brunnipes* | (Fabricius, 1793) | Staphylinidae | Macrofauna predators |
| Coleoptera | *Liogluta longiuscula* | (Gravenhorst, 1802) | Staphylinidae | Macrofauna predators |
| Coleoptera | *Luperus luperus* | (Sulzer, 1776) | Chrysomelidae | Macrofauna herbivores |
| Coleoptera | *Malthinus seriepunctatus* | Kiesenwetter, 1852 | Cantharidae | Macrofauna predators |
| Coleoptera | *Microlestes minutulus* | (Goeze, 1777) | Carabidae | Macrofauna predators |
| Coleoptera | *Mocyta fungi* | (Gravenhorst, 1806) | Staphylinidae | Macrofauna predators |
| Coleoptera | *Molops elatus* | (Fabricius, 1801) | Carabidae | Macrofauna predators |
| Coleoptera | *Molops piceus* | (Panzer, 1793) | Carabidae | Macrofauna predators |
| Coleoptera | *Mycetoporus mulsanti* | Ganglbauer, 1895 | Staphylinidae | Macrofauna predators |
| Coleoptera | *Nargus anisotomoides* | (Spence, 1813) | Leiodidae | Macrofauna decomposers |
| Coleoptera | *Nargus wilkinii* | (Spence, 1813) | Leiodidae | Macrofauna decomposers |
| Coleoptera | *Neobisnius villosulus* | (Stephens, 1833) | Staphylinidae | Macrofauna predators |
| Coleoptera | *Notiophilus biguttatus* | (Fabricius, 1779) | Carabidae | Macrofauna predators |
| Coleoptera | *Notiophilus rufipes* | Curtis, 1829 | Carabidae | Macrofauna predators |
| Coleoptera | *Olophrum piceum* | (Gyllenhal, 1810) | Staphylinidae | Macrofauna predators |
| Coleoptera | *Omonadus floralis* | (Linnaeus, 1758) | Anthicidae | Macrofauna decomposers |
| Coleoptera | *Oodes helopioides* | (Fabricius, 1792) | Carabidae | Macrofauna predators |
| Coleoptera | *Othius punctulatus* | (Goeze, 1777) | Staphylinidae | Macrofauna predators |
| Coleoptera | *Othius subuliformis* | Stephens, 1833 | Staphylinidae | Macrofauna predators |
| Coleoptera | *Oxypoda annularis* | (Mannerheim, 1830) | Staphylinidae | Macrofauna predators |
| Coleoptera | *Panagaeus bipustulatus* | (Fabricius, 1775) | Carabidae | Macrofauna predators |
| Coleoptera | *Philonthus carbonarius* | (Gravenhorst, 1802) | Staphylinidae | Macrofauna predators |
| Coleoptera | *Phyllobius glaucus* | (Scopoli, 1763) | Curculionidae | Macrofauna herbivores |
| Coleoptera | *Phyllobius oblongus* | (Linnaeus, 1758) | Curculionidae | Macrofauna herbivores |
| Coleoptera | *Phyllobius pyri* | Schoenherr, 1826 | Curculionidae | Macrofauna herbivores |
| Coleoptera | *Plectophloeus fischeri* | (Aubé, 1833) | Staphylinidae | Macrofauna predators |
| Coleoptera | *Pterostichus burmeisteri* | Heer, 1838 | Carabidae | Macrofauna predators |
| Coleoptera | *Pterostichus chameleon* | (Motschulsky, 1866) | Carabidae | Macrofauna predators |
| Coleoptera | *Pterostichus gracilior* | LeConte, 1873 | Carabidae | Macrofauna predators |
| Coleoptera | *Pterostichus oblongopunctatus* | (Fabricius, 1787) | Carabidae | Macrofauna predators |
| Coleoptera | *Pterostichus strenuus* | (Csiki, 1930 | Carabidae | Macrofauna predators |
| Coleoptera | *Rhagonycha lignosa* | (Müller, 1764) | Cantharidae | Macrofauna predators |
| Coleoptera | *Rhagonycha nigriventris* | Motschulsky, 1860 | Cantharidae | Macrofauna predators |
| Coleoptera | *Rhynchaeninae* sp.1 |  | Curculionidae | Macrofauna herbivores |
| Coleoptera | *Rhynchaenus fagi* | A. Klima, 1935 | Curculionidae | Macrofauna herbivores |
| Coleoptera | *Rugilus rufipes* | Germar, 1836 | Staphylinidae | Macrofauna predators |
| Coleoptera | *Scydmaenidae* sp.1 |  | Scydmaenidae | Macrofauna predators |
| Coleoptera | *Scydmaenidae* sp.2 |  | Scydmaenidae | Macrofauna predators |
| Coleoptera | *Sepedophilus testaceus* | (Fabricius, 1792) | Staphylinidae | Macrofauna predators |
| Coleoptera | *Staphylinidae* sp.1 |  | Staphylinidae | Macrofauna predators |
| Coleoptera | *Stenus clavicornis* | Gravenhorst, 1802 | Staphylinidae | Macrofauna predators |
| Coleoptera | *Stenus fuscicornis* | Erichson, 1840 | Staphylinidae | Macrofauna predators |
| Coleoptera | *Stenus humilis* | Erichson, 1839 | Staphylinidae | Macrofauna predators |
| Coleoptera | *Stenus impressus* | Germar, 1824 | Staphylinidae | Macrofauna predators |
| Coleoptera | *Stenus mendicus* | Erichson, 1840 | Staphylinidae | Macrofauna predators |
| Coleoptera | *Synuchus vivalis* | (Illiger, 1798) | Carabidae | Macrofauna predators |
| Coleoptera | *Tachinus scapularis* | Stephens, 1832 | Staphylinidae | Macrofauna predators |
| Coleoptera | *Tachyporus obtusus* | (Linnaeus, 1767) | Staphylinidae | Macrofauna predators |
| Coleoptera | *Tenebrionidae* sp.1 |  | Tenebrionidae | Macrofauna decomposers |
| Coleoptera | *Trechus nigrinus* | Putzeys, 1847 | Carabidae | Macrofauna predators |
| Coleoptera | *Trimium brevicorne* | (Reichenbach, 1816) | Staphylinidae | Macrofauna predators |
| Coleoptera | *Xantholinus laevigatus* | Jacobsen, 1849 | Staphylinidae | Macrofauna predators |
| Coleoptera | *Xantholinus tricolor* | (Fabricius, 1787) | Staphylinidae | Macrofauna predators |
| Collembola | *Allacma fusca* | (Linnaeus, 1758) | Sminthuridae | Mesofauna decomposers |
| Collembola | *Anurida pygmaea* | (Börner, 1901) | Neanuridae | Mesofauna predators |
| Collembola | *Arrhopalites pygmaeus* | (Wankel, 1860) | Arrhopalitidae | Mesofauna decomposers |
| Collembola | *Ballistura hankoi* | (Stach, 1959) | Isotomidae | Mesofauna decomposers |
| Collembola | *Brachystomella parvula* | (Schaeffer, 1896) | Brachystomellidae | Mesofauna decomposers |
| Collembola | *Ceratophysella armata* | (Nicolet, 1841) | Hypogastruridae | Mesofauna decomposers |
| Collembola | *Ceratophysella denticulata* | (Bagnall, 1941) | Hypogastruridae | Mesofauna decomposers |
| Collembola | *Ceratophysella gibbosa* | (Bagnall, 1940) | Hypogastruridae | Mesofauna decomposers |
| Collembola | *Ceratophysella succinea* | (Gisin, 1949) | Hypogastruridae | Mesofauna decomposers |
| Collembola | *Deuterosminthurus pallipes* | (Bourlet, 1842) | Bourletiellidae | Mesofauna decomposers |
| Collembola | *Dicyrtoma fuscus* | (Lubbock, 1873) | Dicyrtomidae | Mesofauna decomposers |
| Collembola | *Dicyrtomina ornata* | (Nicolet, 1842) | Dicyrtomidae | Mesofauna decomposers |
| Collembola | *Entomobrya* cf. *multifasciatus* | (Tullberg, 1871) | Entomobryidae | Mesofauna decomposers |
| Collembola | *Entomobrya corticalis* | (Nicolet, 1842) | Entomobryidae | Mesofauna decomposers |
| Collembola | *Entomobrya marginata* | Tullberg, 1871 | Entomobryidae | Mesofauna decomposers |
| Collembola | *Entomobrya quinquelineata* | Börner, 1901 | Entomobryidae | Mesofauna decomposers |
| Collembola | *Folsomia brevicauda* | Agrell, 1939 | Isotomidae | Mesofauna decomposers |
| Collembola | *Folsomia fimetaria* | (Linnaeus, 1758) | Isotomidae | Mesofauna decomposers |
| Collembola | *Folsomia ksenemani* | Stach, 1947 | Isotomidae | Mesofauna decomposers |
| Collembola | *Folsomia litsteri* | Bagnall, 1939 | Isotomidae | Mesofauna decomposers |
| Collembola | *Folsomia quadrioculata* | (Tullberg, 1871) | Isotomidae | Mesofauna decomposers |
| Collembola | *Folsomia spinosa* | Kseneman, 1936 | Isotomidae | Mesofauna decomposers |
| Collembola | *Friesea claviseta* | Axelson, 1900 | Neanuridae | Mesofauna predators |
| Collembola | *Friesea mirabilis* | (Tullberg, 1871) | Neanuridae | Mesofauna predators |
| Collembola | *Friesea truncata* | Cassagnau, 1958 | Neanuridae | Mesofauna predators |
| Collembola | *Gisinianus flammeolus* | (Gisin, 1957) | Katiannidae | Mesofauna decomposers |
| Collembola | *Hypogastrura burkilli* | (Bagnall, 1940) | Hypogastruridae | Mesofauna decomposers |
| Collembola | *Hypogastrura purpurescens* | (Lubbock, 1967) | Hypogastruridae | Mesofauna decomposers |
| Collembola | *Isotoma hiemalis* | Schoett, 1893 | Isotomidae | Mesofauna decomposers |
| Collembola | *Isotoma notabilis* | Schaeffer, 1896 | Isotomidae | Mesofauna decomposers |
| Collembola | *Isotoma violacea* | Tullberg, 1876 | Isotomidae | Mesofauna decomposers |
| Collembola | *Isotomiella minor* | (Schaeffer, 1896) | Isotomidae | Mesofauna decomposers |
| Collembola | *Isotomurus palustris* | (Müller, 1776) | Isotomidae | Mesofauna decomposers |
| Collembola | *Lepidocyrtus curvicollis* | Bourlet, 1839 | Entomobryidae | Mesofauna decomposers |
| Collembola | *Lepidocyrtus cyaneus* | Tullberg, 1871 | Entomobryidae | Mesofauna decomposers |
| Collembola | *Lepidocyrtus lanuginosus* | (Gmelin, 1788) | Entomobryidae | Mesofauna decomposers |
| Collembola | *Lepidocyrtus lignorum* | (Fabricius, 1793) | Entomobryidae | Mesofauna decomposers |
| Collembola | *Lipothrix lubbocki* | (Tullberg, 1872) | Sminthurida | Mesofauna decomposers |
| Collembola | *Megalothorax minimus* | Willem, 1900 | Neelidae | Mesofauna decomposers |
| Collembola | *Mesaphorura italica* | (Rusek, 1971) | Tullbergiidae | Mesofauna decomposers |
| Collembola | *Mesaphorura jarmilae* | Rusek, 1982 | Tullbergiidae | Mesofauna decomposers |
| Collembola | *Mesaphorura sylvatica* | (Rusek, 1971) | Tullbergiidae | Mesofauna decomposers |
| Collembola | *Mesaphorura tenuisensillata* | Rusek, 1974 | Tullbergiidae | Mesofauna decomposers |
| Collembola | *Metaphorura affinis* | (Börner, 1903) | Onychiuridae | Mesofauna decomposers |
| Collembola | *Micranurida* cf. *sensillata* | (Gisin, 1953) | Neanuridae | Mesofauna predators |
| Collembola | *Micranurida forsslundi* | Gisin, 1949 | Neanuridae | Mesofauna predators |
| Collembola | *Micranurida granulata* | (Agrell, 1943) | Neanuridae | Mesofauna predators |
| Collembola | *Micraphorura absoloni* | (Börner, 1901) | Onychiuridae | Mesofauna decomposers |
| Collembola | *Mucrosomia garretti* | (Bagnall, 1939) | Isotomidae | Mesofauna decomposers |
| Collembola | *Neanura muscorum* | (Templeton, 1835) | Neanuridae | Mesofauna predators |
| Collembola | *Neelus minutus* | (Folsom, 1901) | Neelidae | Mesofauna decomposers |
| Collembola | *Neonaphorura duboscqi* | (Denis, 1932) | Onychiuridae | Mesofauna decomposers |
| Collembola | *Neotullbergia ramicuspis* | (Gisin, 1953) | Onychiuridae | Mesofauna decomposers |
| Collembola | *Oncopodura crassicornis* | Shoebotham, 1911 | Oncopoduridae | Mesofauna decomposers |
| Collembola | *Onychiurus armatus* | (Tullberg, 1869) | Onychiuridae | Mesofauna decomposers |
| Collembola | *Onychiurus inermis* | (Tullberg, 1871) | Onychiuridae | Mesofauna decomposers |
| Collembola | *Orchesella bifasciata* | Nicolet, 1842 | Entomobryidae | Mesofauna decomposers |
| Collembola | *Orchesella* cf. *flavescens* | (Bourlet, 1839) | Entomobryidae | Mesofauna decomposers |
| Collembola | *Paratullbergia macdougalli* | Bagnall, 1936 | Onychiuridae | Mesofauna predators |
| Collembola | *Proisotoma minima* | (Absolon, 1901) | Isotomidae | Mesofauna decomposers |
| Collembola | *Proisotoma minuta* | (Tullberg, 1871) | Isotomidae | Mesofauna decomposers |
| Collembola | *Protaphorura aurantiaca* | (Ridley, 1880) | Onychiuridae | Mesofauna decomposers |
| Collembola | *Protaphorura fimata* | (Gisin, 1952) | Onychiuridae | Mesofauna decomposers |
| Collembola | *Protaphorura quadriocellata* | (Gisin, 1947) | Onychiuridae | Mesofauna decomposers |
| Collembola | *Pseudachorutes dubius* | Krausbauer, 1898 | Neanuridae | Mesofauna predators |
| Collembola | *Pseudachorutes subcrassus* | Tullberg, 1871 | Neanuridae | Mesofauna predators |
| Collembola | *Pseudanurophorus binoculatus* | (Kseneman, 1934) | Isotomidae | Mesofauna decomposers |
| Collembola | *Pseudosinella alba* | (Packard, 1873) | Entomobryidae | Mesofauna decomposers |
| Collembola | *Pseudosinella decipiens* | Denis, 1924 | Entomobryidae | Mesofauna decomposers |
| Collembola | *Pseudosinella denisi* | Gisin, 1954 | Entomobryidae | Mesofauna decomposers |
| Collembola | *Pseudosinella immaculata* | (Lie-Pettersen, 1897) | Entomobryidae | Mesofauna decomposers |
| Collembola | *Sminthurides pumilus* | (Krausbauer, 1898) | Sminthurididae | Mesofauna decomposers |
| Collembola | *Sminthurinus aureus* | (Lubbock, 1862) | Katiannidae | Mesofauna decomposers |
| Collembola | *Sminthurinus niger* | (Lubbock, 1868) | Katiannidae | Mesofauna decomposers |
| Collembola | *Sminthurus viridis* | (Linnaeus, 1758) | Sminthuridae | Mesofauna decomposers |
| Collembola | *Stenaphorura denisi* | Bagnall, 1935 | Tullbergiidae | Mesofauna decomposers |
| Collembola | *Supraphorura furcifer* | (Börner, 1901) | Onychiuridae | Mesofauna decomposers |
| Collembola | *Tomocerus baudoti* | Denis, 1932 | Tomoceridae | Mesofauna decomposers |
| Collembola | *Tomocerus flavescens* | Tullberg, 1871 | Tomoceridae | Mesofauna decomposers |
| Collembola | *Tomocerus minor* | (Lubbock, 1862) | Tomoceridae | Mesofauna decomposers |
| Collembola | *Tomocerus minutus* | Folsom, 1901 | Tomoceridae | Mesofauna decomposers |
| Collembola | *Tomocerus vulgaris* | (Tullberg, 1871) | Tomoceridae | Mesofauna decomposers |
| Collembola | *Tullbergia callipygos* | Börner, 1902 | Tullbergiidae | Mesofauna predators |
| Collembola | *Tullbergia macrochaeta* | (Rusek, 1976) | Tullbergiidae | Mesofauna decomposers |
| Collembola | *Tullbergia quadrispina* | (Börner, 1901) | Tullbergiidae | Mesofauna decomposers |
| Collembola | *Tullbergia yosii* | (Rusek, 1967) | Tullbergiidae | Mesofauna decomposers |
| Collembola | *Willemia anophthalma* | Börner, 1901 | Hypogastruridae | Mesofauna decomposers |
| Collembola | *Willemia aspinata* | Stach, 1949 | Hypogastruridae | Mesofauna decomposers |
| Collembola | *Xenylla grisea* | Axelson, 1900 | Hypogastruridae | Mesofauna decomposers |
| Diplopoda | *Allajulus nitidus* | (Verhoeff, 1891) | Julidae | Macrofauna decomposers |
| Diplopoda | *Brachyiulus pusillus* | (Bosc, 1792) | Julidae | Macrofauna decomposers |
| Diplopoda | *Chordeuma sylvestre* | C. L. Koch, 1847 | Chordeumatidae | Macrofauna decomposers |
| Diplopoda | *Eurypleuromeris conspersa* | C. L. Koch, 1847 | Glomeridae | Macrofauna decomposers |
| Diplopoda | *Geoglomeris* cf. *subterranea* | Verhoeff, 1908 | Glomeridae | Macrofauna decomposers |
| Diplopoda | *Glomeris connexa* | C. L. Koch, 1847 | Glomeridae | Macrofauna decomposers |
| Diplopoda | *Glomeris hexasticha* | Brandt, 1833 | Glomeridae | Macrofauna decomposers |
| Diplopoda | *Glomeris marginata* | (Berlese, 1892) | Glomeridae | Macrofauna decomposers |
| Diplopoda | *Glomeris undulata* | C. L. Koch, 1844 | Glomeridae | Macrofauna decomposers |
| Diplopoda | *Megaphyllum projectum* | Verhoeff, 1894 | Julidae | Macrofauna decomposers |
| Diplopoda | *Melogona* cf. *voigti* | (Verhoeff, 1899) | Chordeumatidae | Macrofauna decomposers |
| Diplopoda | *Polydesmus angustus* | Latzel, 1884 | Polydesmidae | Macrofauna decomposers |
| Diplopoda | *Polydesmus complanatus* | (Linnaeus, 1761) | Polydesmidae | Macrofauna decomposers |
| Diplopoda | *Polydesmus denticulatus* | Le Guillou, 1841 | Polydesmidae | Macrofauna decomposers |
| Diplopoda | *Polydesmus inconstans* | Latzel, 1883 | Polydesmidae | Macrofauna decomposers |
| Diplopoda | *Polyxenus lagura* | (Linnaeus, 1758) | Polyxenidae | Macrofauna decomposers |
| Diplopoda | *Proteroiulus fuscus* | (Am Stein, 1857) | Blaniulidae | Macrofauna decomposers |
| Diplopoda | *Tachypodoiulus niger* | (Leach, 1814) | Julidae | Macrofauna decomposers |
| Diplopoda | *Unciger foetidus* | (C. L. Koch, 1838) | Julidae | Macrofauna decomposers |
| Diplura | *Campodea* cf. *plusiochaeta* | (Silvestri, 1912) | Campodeidae | Macrofauna predators |
| Gastropoda | *Aegopinella nitens* | (Michaud, 1831) | Gastrodontidae | Macrofauna decomposers |
| Gastropoda | *Aegopinella nitidula* | (Draparnaud, 1805) | Gastrodontidae | Macrofauna decomposers |
| Gastropoda | *Aegopinella pura* | (Alder, 1830) | Gastrodontidae | Macrofauna decomposers |
| Gastropoda | *Arianta arbustorum* | (Linnaeus, 1758) | Helicidae | Macrofauna herbivores |
| Gastropoda | *Arion ater* | (Linnaeus, 1758) | Arionidae | Macrofauna decomposers |
| Gastropoda | *Arion fuscus* | (O. F. Müller, 1774) | Arionidae | Macrofauna decomposers |
| Gastropoda | *Arion intermedius* | Normand, 1852 | Arionidae | Macrofauna decomposers |
| Gastropoda | *Arion silvaticus* | Lohmander, 1937 | Arionidae | Macrofauna decomposers |
| Gastropoda | *Azeca goodalli* | (A. Férussac, 1821) | Azecidae | Macrofauna decomposers |
| Gastropoda | *Cepaea hortensis* | (O. F. Müller, 1774) | Helicidae | Macrofauna herbivores |
| Gastropoda | *Cepaea nemoralis* | (Linnaeus, 1758) | Helicidae | Macrofauna herbivores |
| Gastropoda | *Clausilia bidentata* | (Strøm, 1765) | Clausiliidae | Macrofauna decomposers |
| Gastropoda | *Cochlicopa lubrica* | (O. F. Müller, 1774) | Cochlicopidae | Macrofauna decomposers |
| Gastropoda | *Cochlodina laminata* | (Montagu, 1803) | Clausiliidae | Macrofauna decomposers |
| Gastropoda | *Discus rotundatus* | (O. F. Müller, 1774) | Discidae | Macrofauna decomposers |
| Gastropoda | *Ena montana* | (Draparnaud, 1801) | Enidae | Macrofauna decomposers |
| Gastropoda | *Euconulus fulvus* | (O. F. Müller, 1774) | Euconulidae | Macrofauna decomposers |
| Gastropoda | *Helicigona lapicida* | (Linnaeus, 1758) | Helicidae | Macrofauna decomposers |
| Gastropoda | *Helicodonta obvoluta* | (O. F. Müller, 1774) | Helicodontidae | Macrofauna herbivores |
| Gastropoda | *Lehmannia marginata* | (O. F. Müller, 1774) | Limacidae | Macrofauna decomposers |
| Gastropoda | *Limax cinereoniger* | Wolf, 1803 | Limacidae | Macrofauna decomposers |
| Gastropoda | *Macrogastra ventricosa* | (Draparnaud, 1801) | Clausiliidae | Macrofauna decomposers |
| Gastropoda | *Monachoides incarnatus* | (O. F. Müller, 1774) | Hygromiidae | Macrofauna decomposers |
| Gastropoda | *Perpolita hammonis* | (Strøm, 1765) | Gastrodontidae | Macrofauna decomposers |
| Gastropoda | *Trichia striolata* | (C. Pfeiffer, 1828) | Hygromiidae | Macrofauna decomposers |
| Gastropoda | *Vitrea diaphana* | (S. Studer, 1820) | Pristilomatidae | Macrofauna decomposers |
| Isopoda | *Armadillidium opacum* | (C. L. Koch, 1841) | Armadillidiidae | Macrofauna decomposers |
| Isopoda | *Haplophthalmus mengii* | (Zaddach, 1844) | Trichoniscidae | Macrofauna decomposers |
| Isopoda | *Ligidium hypnorum* | (Cuvier, 1792) | Ligiidae | Macrofauna decomposers |
| Isopoda | *Oniscus asellus* | Cuvier, 1792 | Oniscidae | Macrofauna decomposers |
| Isopoda | *Philoscia muscorum* | (Scopoli, 1763) | Philosciidae | Macrofauna decomposers |
| Isopoda | *Porcellio dilatatus* | Brandt, 1831 | Porcellionidae | Macrofauna decomposers |
| Isopoda | *Porcellio montanus* | Budde-Lund, 1885 | Porcellionidae | Macrofauna decomposers |
| Isopoda | *Porcellio spinicornis* | Say, 1818 | Porcellionidae | Macrofauna decomposers |
| Isopoda | *Porcellium conspersum* | (C. L. Koch, 1841) | Trachelipodidae | Macrofauna decomposers |
| Isopoda | *Trachelipus rathkii* | (Brandt, 1833) | Trachelipodidae | Macrofauna decomposers |
| Isopoda | *Trachelipus ratzeburgii* | (Brandt, 1833) | Trachelipodidae | Macrofauna decomposers |
| Isopoda | *Trichoniscus pusillus* | Brandt, 1833 | Trichoniscidae | Macrofauna decomposers |
| Isopoda | *Trichoniscus pygmaeus* | G. O. Sars, 1898 | Trichoniscidae | Macrofauna decomposers |
| Lumbricidae | *Allolobophora longa* | Ude, 1885 | Lumbricidae | Macrofauna decomposers |
| Lumbricidae | *Aporrectodea caliginosa* | (Savigny, 1826) | Lumbricidae | Macrofauna decomposers |
| Lumbricidae | *Aporrectodea rosea* | (Savigny, 1826) | Lumbricidae | Macrofauna decomposers |
| Lumbricidae | *Bimastos rubidus* | (Savigny, 1826) | Lumbricidae | Macrofauna decomposers |
| Lumbricidae | *Dendrobaena octaedra* | (Savigny, 1826) | Lumbricidae | Macrofauna decomposers |
| Lumbricidae | *Enterion castaneum* | Savigny, 1826 | Lumbricidae | Macrofauna decomposers |
| Lumbricidae | *Enterion pygmaeum* | Savigny, 1826 | Lumbricidae | Macrofauna decomposers |
| Lumbricidae | *Enterion tyrtaeum* | Savigny, 1826 | Lumbricidae | Macrofauna decomposers |
| Lumbricidae | *Lumbricus rubellus* | Hoffmeister, 1843 | Lumbricidae | Macrofauna decomposers |
| Lumbricidae | *Lumbricus terrestris* | Linnaeus, 1758 | Lumbricidae | Macrofauna decomposers |
| Mesostigmata | *Amblyseius* cf. *nemorivagus* | Athias-Henriot, 1961 | Phytoseiidae | Mesofauna predators |
| Mesostigmata | *Amblyseius similifloridanus* | (Hirschmann, 1962) | Phytoseiidae | Mesofauna predators |
| Mesostigmata | *Asca bicornis* | (Canestrini & Fanzago, 1887) | Ascidae | Mesofauna predators |
| Mesostigmata | *Cilliba athiasae* | (Hirschmann & Zirngiebl-Nicol, 1969) | Uropodidae | Mesofauna predators |
| Mesostigmata | *Cilliba cassidea* | (Hermann, 1804) | Uropodidae | Mesofauna predators |
| Mesostigmata | *Cornodendrolaelaps cf. cornutulus* | (W. Hirschmann, 1960) | Digamasellidae | Mesofauna predators |
| Mesostigmata | *Dinychus perforatus* | P. Kramer, 1886 | Dinychidae | Mesofauna predators |
| Mesostigmata | *Discourella shcherbakae* | Hirschmann, 1972 | Uropodidae | Mesofauna predators |
| Mesostigmata | *Epicrius canestrinii* | Haller, 1881 | Epicriidae | Mesofauna predators |
| Mesostigmata | *Epicrius* cf. *spinituberculatus* | Evans, 1955 | Epicriidae | Mesofauna predators |
| Mesostigmata | *Epicrius schusteri* | Blaszak & Alberti, 1989 | Epicriidae | Mesofauna predators |
| Mesostigmata | *Gaeolaelaps aculeifer* | (G. Canestrini, 1884) | Laelapidae | Mesofauna predators |
| Mesostigmata | *Gamasellodes bicolor* | (A. Berlese, 1918) | Ascidae | Mesofauna predators |
| Mesostigmata | *Geholaspis aeneus* | Krauss, 1970 | Macrochelidae | Mesofauna predators |
| Mesostigmata | *Geholaspis longispinosus* | (P. Kramer, 1876) | Macrochelidae | Mesofauna predators |
| Mesostigmata | *Geholaspis mandibularis* | (Berlese, 1904) | Macrochelidae | Mesofauna predators |
| Mesostigmata | *Holoparasitus stramenti* | Karg, 1971 | Parasitidae | Mesofauna predators |
| Mesostigmata | *Lasioseius lawrencei* | (G. O. Evans, 1958) | Ascidae | Mesofauna predators |
| Mesostigmata | *Lasioseius magnanalis* | (Evans, 1958) | Ascidae | Mesofauna predators |
| Mesostigmata | *Leioseius elongatus* | Evans, 1958 | Ascidae | Mesofauna predators |
| Mesostigmata | *Leitneria granulata* | (Halbert, 1923) | Halolaelapidae | Mesofauna predators |
| Mesostigmata | *Leptogamasus suecicus* | Trägårdh, 1936 | Parasitidae | Mesofauna predators |
| Mesostigmata | *Lysigamasus* cf .*arcuatus* | (Dielmannn, 1991 i.l.) | Parasitidae | Mesofauna predators |
| Mesostigmata | *Lysigamasus* cf. *rostriforceps* | Athias-Henriot, 1967 | Parasitidae | Mesofauna predators |
| Mesostigmata | *Lysigamasus* cf. *wasmanni* | (Oudemans, 1902) | Parasitidae | Mesofauna predators |
| Mesostigmata | *Lysigamasus digitulus* | Karg, 1963 | Parasitidae | Mesofauna predators |
| Mesostigmata | *Lysigamasus jugincola* | Athias-Henriot, 1967 | Parasitidae | Mesofauna predators |
| Mesostigmata | *Lysigamasus minorleitneriae* | Athias-Henriot, 1967 | Parasitidae | Mesofauna predators |
| Mesostigmata | *Macrocheles* cf. *opacus* subsp. *aciculatus* | Berlese, 1918 | Macrochelidae | Mesofauna predators |
| Mesostigmata | *Macrocheles dentatus* | (Evans & Browning, 1956) | Macrochelidae | Mesofauna predators |
| Mesostigmata | *Macrocheles montanus* | (C. Willmann, 1951) | Macrochelidae | Mesofauna predators |
| Mesostigmata | *Macrocheles opacus* | (C. L. Koch, 1839) | Macrochelidae | Mesofauna predators |
| Mesostigmata | *Pachylaelaps bellicosus* | Berlese, 1920 | Pachylaelapidae | Mesofauna predators |
| Mesostigmata | *Pachylaelaps* cf. *vexillifer* | Willmann, 1961 | Pachylaelapidae | Mesofauna predators |
| Mesostigmata | *Pachylaelaps fuscinuliger* | Berlese, 1921 | Pachylaelapidae | Mesofauna predators |
| Mesostigmata | *Pachylaelaps laeuchlii* | Schweitzer, 1922 | Pachylaelapidae | Mesofauna predators |
| Mesostigmata | *Pachylaelaps longisetus* | Halbert, 1915 | Pachylaelapidae | Mesofauna predators |
| Mesostigmata | *Pachylaelaps regularis* | Berlese, 1920 | Pachylaelapidae | Mesofauna predators |
| Mesostigmata | *Pachylaelaps tesselatus* | Berlese, 1920 | Pachylaelapidae | Mesofauna predators |
| Mesostigmata | *Pachyseius angustus* | Hyatt, 1956 | Pachylaelapidae | Mesofauna predators |
| Mesostigmata | *Pachyseius humeralis* | Berlese, 1910 | Pachylaelapidae | Mesofauna predators |
| Mesostigmata | *Paragamasus lapponicus* | (Trägårdh, 1910) | Parasitidae | Mesofauna predators |
| Mesostigmata | *Paragamasus parrunciger* | (Bhattacharyya, 1963) | Parasitidae | Mesofauna predators |
| Mesostigmata | *Paragamasus truncus* | Athias-Henriot, 1967 | Parasitidae | Mesofauna predators |
| Mesostigmata | *Paragamasus vagabundus* | (Karg, 1968) | Parasitidae | Mesofauna predators |
| Mesostigmata | *Pergamasus celticus* | (Bhattacharyya, 1963) | Parasitidae | Mesofauna predators |
| Mesostigmata | *Pergamasus* cf. *conus* | (Karg, 1971) | Parasitidae | Mesofauna predators |
| Mesostigmata | *Pergamasus* cf. *runcatellus* | (Berlese, 1906) | Parasitidae | Mesofauna predators |
| Mesostigmata | *Pergamasus* cf. *tectegynellus* | Athias-Henriot, 1967 | Parasitidae | Mesofauna predators |
| Mesostigmata | *Pergamasus cornutus* | (Schweitzer, 1961) | Parasitidae | Mesofauna predators |
| Mesostigmata | *Pergamasus crassipes* | (Linnaeus, 1758) | Parasitidae | Mesofauna predators |
| Mesostigmata | *Pergamasus misellus* | Athias-Henriot, 1967 | Parasitidae | Mesofauna predators |
| Mesostigmata | *Pergamasus norvegicus* | (Berlese, 1905) | Parasitidae | Mesofauna predators |
| Mesostigmata | *Pergamasus puerilis* | (Karg, 1963) | Parasitidae | Mesofauna predators |
| Mesostigmata | *Pergamasus quisquiliarum* | (Canestrini, 1882) | Parasitidae | Mesofauna predators |
| Mesostigmata | *Pergamasus runcatellus* | (Berlese, 1906) | Parasitidae | Mesofauna predators |
| Mesostigmata | *Pergamasus septentrionalis* | (Oudemans, 1902) | Parasitidae | Mesofauna predators |
| Mesostigmata | *Pergamasus solitarius* | Karg, 1968 | Parasitidae | Mesofauna predators |
| Mesostigmata | *Prozercon* cf. *traegardhi* | (Halbert, 1923) | Zerconidae | Mesofauna predators |
| Mesostigmata | *Prozercon fimbriatus* | (C. L. Koch, 1839) | Zerconidae | Mesofauna predators |
| Mesostigmata | *Prozercon kochi* | Sellnick, 1943 | Zerconidae | Mesofauna predators |
| Mesostigmata | *Pseudoparasitus placentulus* | (Berlese, 1887) | Laelapidae | Mesofauna predators |
| Mesostigmata | *Rhodacarellus kreuzi* | Karg, 1965 | Rhodacaridae | Mesofauna predators |
| Mesostigmata | *Rhodacarus agrestis* | Karg, 1971 | Rhodacaridae | Mesofauna predators |
| Mesostigmata | *Rhodacarus coronatus* | Berlese, 1920 | Rhodacaridae | Mesofauna predators |
| Mesostigmata | *Trachytes aegrota* | (C. L. Koch, 1841) | Polyaspididae | Mesofauna predators |
| Mesostigmata | *Trachytes pauperior* | Berlese, 1914 | Polyaspididae | Mesofauna predators |
| Mesostigmata | *Trichouropoda obscura* | (C. L. Koch, 1836) | Trematuridae | Mesofauna predators |
| Mesostigmata | *Trichouropoda ovalis* | (C. L. Koch, 1839) | Trematuridae | Mesofauna predators |
| Mesostigmata | *Urodiaspis tecta* | (P. Kramer, 1876) | Urodiaspididae | Mesofauna predators |
| Mesostigmata | *Uropoda* cf. *splendida* | P. Kramer, 1882 | Uropodidae | Mesofauna predators |
| Mesostigmata | *Uropoda minima* | P. Kramer, 1882 | Uropodidae | Mesofauna predators |
| Mesostigmata | *Uroseius cylindricus* | (Berlese, 1916) | Polyaspididae | Mesofauna predators |
| Mesostigmata | *Veigaia agilis* | (Berlese, 1916) | Veigaiidae | Mesofauna predators |
| Mesostigmata | *Veigaia cerva* | (P. Kramer, 1876) | Veigaiidae | Mesofauna predators |
| Mesostigmata | *Veigaia* cf. *mollis* | Karg, 1971 | Veigaiidae | Mesofauna predators |
| Mesostigmata | *Veigaia* cf. *propinqua* | Willmann, 1936 | Veigaiidae | Mesofauna predators |
| Mesostigmata | *Veigaia exigua* | (Berlese, 1916) | Veigaiidae | Mesofauna predators |
| Mesostigmata | *Veigaia kochi* | (Trägårdh, 1901) | Veigaiidae | Mesofauna predators |
| Mesostigmata | *Veigaia nemorensis* | (C. L. Koch, 1839) | Veigaiidae | Mesofauna predators |
| Mesostigmata | *Veigaia planicola* | (Berlese, 1892) | Veigaiidae | Mesofauna predators |
| Mesostigmata | *Vulgarogamasus kraepelini* | (Berlese, 1905) | Parasitidae | Mesofauna predators |
| Mesostigmata | *Vulgarogamasus remberti* | (Oudemans, 1912) | Parasitidae | Mesofauna predators |
| Mesostigmata | *Zercon* cf. *peltatus* | C. L. Koch, 1836 | Zerconidae | Mesofauna predators |
| Mesostigmata | *Zercon* cf. *romagniolus* | Sellnick, 1944 | Zerconidae | Mesofauna predators |
| Mesostigmata | *Zercon* cf. *triangularis* | C. L. Koch, 1836 | Zerconidae | Mesofauna predators |
| Mesostigmata | *Zercon gurensis* | Mihelcic, 1962 | Zerconidae | Mesofauna predators |
| Mesostigmata | *Zerconopsis remiger* | (P. Kramer, 1876) | Ascidae | Mesofauna predators |
| Opiliones | *Anelasmocephalus cambridgei* | (Westwood, 1847) | Trogulidae | Macrofauna predators |
| Opiliones | *Trogulus nepaeformis* | (Scopoli, 1763) | Trogulidae | Macrofauna predators |
| Opiliones | *Trogulus tricarinatus* | (Linnaeus, 1767) | Trogulidae | Macrofauna predators |
| Oribatida | *Achipteria coleoptrata* | (Linnaeus, 1758) | Achipteriidae | Mesofauna decomposers |
| Oribatida | *Achipteria nitens* | (Nicolet, 1855) | Achipteriidae | Mesofauna decomposers |
| Oribatida | *Acrotritia duplicata* | (Grandjean, 1953) | Euphthiracaridae | Mesofauna decomposers |
| Oribatida | *Adoristes ovatus* | (C. L. Koch, 1840) | Liacaridae | Mesofauna decomposers |
| Oribatida | *Allosuctobelba grandis* | (Paoli, 1908) | Suctobelbidae | Mesofauna predators |
| Oribatida | *Amerus polonicus* | Kulczynski, 1902 | Ameridae | Mesofauna decomposers |
| Oribatida | *Atropacarus striculus* | (C. L. Koch, 1835) | Phthiracaridae | Mesofauna decomposers |
| Oribatida | *Belba corynopus* | (Hermann, 1804) | Damaeidae | Mesofauna decomposers |
| Oribatida | *Berniniella bicarinata* | (Paoli, 1908) | Oppiidae | Mesofauna predators |
| Oribatida | *Berniniella conjuncta* | (Strenzke, 1951) | Oppiidae | Mesofauna predators |
| Oribatida | *Berniniella sigma* | (Strenzke, 1951) | Oppiidae | Mesofauna predators |
| Oribatida | *Carabodes coriaceus* | C. L. Koch, 1835 | Carabodidae | Mesofauna decomposers |
| Oribatida | *Carabodes femoralis* | (Nicolet, 1855) | Carabodidae | Mesofauna decomposers |
| Oribatida | *Carabodes labyrinthicus* | (Michael, 1879) | Carabodidae | Mesofauna decomposers |
| Oribatida | *Carabodes ornatus* | Storkan, 1925 | Carabodidae | Mesofauna decomposers |
| Oribatida | *Carabodes subarcticus* | Trägårdh, 1902 | Carabodidae | Mesofauna decomposers |
| Oribatida | *Cepheus cepheiformis* | (Nicolet, 1855) | Cepheidae | Mesofauna decomposers |
| Oribatida | *Ceratozetes gracilis* | (Michael, 1884) | Ceratozetidae | Mesofauna decomposers |
| Oribatida | *Chamobates cuspidatus* | (Michael, 1884) | Chamobatidae | Mesofauna decomposers |
| Oribatida | *Chamobates pusillus* | (Berlese, 1895) | Chamobatidae | Mesofauna decomposers |
| Oribatida | *Chamobates pusillus* | (Berlese, 1895) | Chamobatidae | Mesofauna decomposers |
| Oribatida | *Chamobates subglobulus* | (Oudemans, 1900) | Chamobatidae | Mesofauna decomposers |
| Oribatida | *Chamobates voigtsi* | (Oudemans, 1902) | Chamobatidae | Mesofauna decomposers |
| Oribatida | *Cultroribula bicultrata* | (Berlese, 1905) | Astegistidae | Mesofauna decomposers |
| Oribatida | *Cymbaeremaeus cymba* | (Nicolet, 1855) | Cymbaeremaeidae | Mesofauna decomposers |
| Oribatida | *Damaeus auritus* | C. L. Koch, 1835 | Damaeidae | Mesofauna decomposers |
| Oribatida | *Damaeus onustus* | C. L. Koch, 1841 | Damaeidae | Mesofauna decomposers |
| Oribatida | *Damaeus riparius* | Nicolet, 1855 | Damaeidae | Mesofauna decomposers |
| Oribatida | *Damaeus setiger* | (Kulczynski, 1902) | Damaeidae | Mesofauna decomposers |
| Oribatida | *Dameobelba minutissima* | (Sellnick, 1920) | Damaeidae | Mesofauna decomposers |
| Oribatida | *Dissorhina ornata* | (Oudemans, 1900) | Oppiidae | Mesofauna predators |
| Oribatida | *Edwardzetes edwardsi* | (Oudemans, 1900) | Ceratozetidae | Mesofauna decomposers |
| Oribatida | *Eulohmannia ribagai* | (Berlese, 1910) | Eulohmanniidae | Mesofauna decomposers |
| Oribatida | *Eupelops hirtus* | (Berlese, 1916) | Phenopelopidae | Mesofauna decomposers |
| Oribatida | *Eupelops plicatus* | (C. L. Koch, 1835) | Phenopelopidae | Mesofauna decomposers |
| Oribatida | *Eupelops torulosus* | (C. L. Koch, 1839) | Phenopelopidae | Mesofauna decomposers |
| Oribatida | *Euzetes globulus* | (Nicolet, 1855) | Ceratozetidae | Mesofauna decomposers |
| Oribatida | *Fosseremus laciniatus* | (Berlese, 1905) | Damaeolidae | Mesofauna decomposers |
| Oribatida | *Fuscozetes setosus* | (C. L. Koch, 1839) | Ceratozetidae | Mesofauna decomposers |
| Oribatida | *Galumna lanceata* | (Oudemans, 1900) | Galumnidae | Mesofauna decomposers |
| Oribatida | *Galumna tarsipennata* | Oudemans, 1913 | Galumnidae | Mesofauna decomposers |
| Oribatida | *Hemileius initialis* | (Berlese, 1908) | Hemileiidae | Mesofauna decomposers |
| Oribatida | *Hermannia gibba* | (C. L. Koch, 1839) | Hermanniidae | Mesofauna decomposers |
| Oribatida | *Hungarobelba pyrenaica* | Miko & Trave, 1996 | Hungarobelbidae | Mesofauna decomposers |
| Oribatida | *Hypochthoniella minutissima* | (Berlese, 1903) | Eniochthoniidae | Mesofauna decomposers |
| Oribatida | *Hypochthonius luteus* | Oudemans, 1917 | Hypochthoniidae | Mesofauna predators |
| Oribatida | *Hypochthonius rufulus* | C. L. Koch, 1835 | Hypochthoniidae | Mesofauna predators |
| Oribatida | *Hypogeoppia dungeri* | Schwalbe, 1995 | Oppiidae | Mesofauna predators |
| Oribatida | *Jugatala angulata* | (C. L. Koch, 1840) | Ceratozetidae | Mesofauna decomposers |
| Oribatida | *Lauroppia falcata* | (Paoli, 1908) | Oppiidae | Mesofauna predators |
| Oribatida | *Lauroppia falcata* | (Paoli, 1908) | Oppiidae | Mesofauna predators |
| Oribatida | *Lauroppia maritima* | (Willmann, 1928) | Oppiidae | Mesofauna predators |
| Oribatida | *Liacarus coracinus* | (C. L. Koch, 1841) | Liacaridae | Mesofauna decomposers |
| Oribatida | *Liacarus subterraneus* | (C. L. Koch, 1844) | Liacaridae | Mesofauna decomposers |
| Oribatida | *Liacarus xylariae* | (Schrank, 1803) | Liacaridae | Mesofauna decomposers |
| Oribatida | *Liebstadia humerata* | Sellnick, 1928 | Liebstadiidae | Mesofauna decomposers |
| Oribatida | *Liebstadia similis* | (Michael, 1888) | Liebstadiidae | Mesofauna decomposers |
| Oribatida | *Metabelba pulverosa* | Strenzke, 1953 | Damaeidae | Mesofauna decomposers |
| Oribatida | *Micreremus brevipes* | (Michael, 1888) | Micreremidae | Mesofauna decomposers |
| Oribatida | *Micreremus gracilior* | Willmann, 1931 | Micreremidae | Mesofauna decomposers |
| Oribatida | *Microppia minus* | (Paoli, 1908) | Oppiidae | Mesofauna predators |
| Oribatida | *Microtritia minima* | (Berlese, 1904) | Euphthiracaridae | Mesofauna decomposers |
| Oribatida | *Murcia nova* | Sellnick, 1928 | Ceratozetidae | Mesofauna decomposers |
| Oribatida | *Nanhermannia elegantula* | Berlese, 1913 | Nanhermanniidae | Mesofauna decomposers |
| Oribatida | *Nanhermannia nana* | (Nicolet, 1855) | Nanhermanniidae | Mesofauna decomposers |
| Oribatida | *Neotrichoppia confinis* | (Paoli, 1908) | Oppiidae | Mesofauna predators |
| Oribatida | *Nothrus palustris* | C. L. Koch, 1839 | Nothridae | Mesofauna decomposers |
| Oribatida | *Nothrus silvestris* | Nicolet, 1855 | Nothridae | Mesofauna decomposers |
| Oribatida | *Ophidiotrichus tectus* | (Michael, 1884) | Oribatellidae | Mesofauna decomposers |
| Oribatida | *Oppiella nova* | (Oudemans, 1902) | Oppiidae | Mesofauna predators |
| Oribatida | *Oppiella obsoleta* | Paoli, 1908 | Oppiidae | Mesofauna predators |
| Oribatida | *Oppiella propinqua* | Mahunka & Mahunka-Papp, 2000 | Oppiidae | Mesofauna predators |
| Oribatida | *Oribatella calcarata* | (C. L. Koch, 1836) | Oribatellidae | Mesofauna decomposers |
| Oribatida | *Oribatula tibialis* | (Nicolet, 1855) | Oribatulidae | Mesofauna decomposers |
| Oribatida | *Pantelozetes paolii* | (Oudemans, 1913) | Oribellidae | Mesofauna decomposers |
| Oribatida | *Parachipteria punctata* | (Nicolet, 1855) | Achipteriidae | Mesofauna decomposers |
| Oribatida | *Pergalumna nervosa* | (Berlese, 1914) | Galumnidae | Mesofauna decomposers |
| Oribatida | *Phthiracarus affinis* | (Hull, 1814) | Phthiracaridae | Mesofauna decomposers |
| Oribatida | *Phthiracarus anonymus* | Grandjean, 1934 | Phthiracaridae | Mesofauna decomposers |
| Oribatida | *Phthiracarus boresetosus* | Jacot, 1930 | Phthiracaridae | Mesofauna decomposers |
| Oribatida | *Phthiracarus* cf. *crenophilus* | Willmann, 1951 | Phthiracaridae | Mesofauna decomposers |
| Oribatida | *Phthiracarus clavatus* | Parry, 1979 | Phthiracaridae | Mesofauna decomposers |
| Oribatida | *Phthiracarus compressus* | Jacot, 1930 | Phthiracaridae | Mesofauna decomposers |
| Oribatida | *Phthiracarus crinitus* | (C. L. Koch, 1841) | Phthiracaridae | Mesofauna decomposers |
| Oribatida | *Phthiracarus ferrugineus* | (C. L. Koch, 1841) | Phthiracaridae | Mesofauna decomposers |
| Oribatida | *Phthiracarus globosus* | (C. L. Koch, 1841) | Phthiracaridae | Mesofauna decomposers |
| Oribatida | *Phthiracarus italicus* | (Oudemans, 1900) | Phthiracaridae | Mesofauna decomposers |
| Oribatida | *Phthiracarus laevigatus* | (C. L. Koch, 1841) | Phthiracaridae | Mesofauna decomposers |
| Oribatida | *Phthiracarus lentulus* | (C. L. Koch, 1841) | Phthiracaridae | Mesofauna decomposers |
| Oribatida | *Phthiracarus longulus* | (C. L. Koch, 1841) | Phthiracaridae | Mesofauna decomposers |
| Oribatida | *Phthiracarus stramineus* | (C. L. Koch, 1841) | Phthiracaridae | Mesofauna decomposers |
| Oribatida | *Pilogalumna crassiclava* | (Berlese, 1914) | Galumnidae | Mesofauna decomposers |
| Oribatida | *Pilogalumna tenuiclava* | (Berlese, 1908) | Galumnidae | Mesofauna decomposers |
| Oribatida | *Platynothrus peltifer* | (C. L. Koch, 1840) | Camisiidae | Mesofauna decomposers |
| Oribatida | *Porobelba spinosa* | (Sellnick, 1920) | Damaeidae | Mesofauna decomposers |
| Oribatida | *Quadroppia hammerae* | Mínguez, Ruiz & Subías, 1985 | Quadroppiidae | Mesofauna decomposers |
| Oribatida | *Quadroppia monstruosa* | Hammer, 1975 | Quadroppiidae | Mesofauna decomposers |
| Oribatida | *Quadroppia quadricarinata* | (Michael, 1885) | Quadroppiidae | Mesofauna decomposers |
| Oribatida | *Rhinoppia fallax* | (Paoli, 1908) | Oppiidae | Mesofauna predators |
| Oribatida | *Rhinoppia subpectinata* | (Oudemans, 1900) | Oppiidae | Mesofauna predators |
| Oribatida | *Scheloribates laevigatus* | (C. L. Koch, 1836) | Scheloribatidae | Mesofauna decomposers |
| Oribatida | *Sphaerozetes piriformis* | (Nicolet, 1855) | Ceratozetidae | Mesofauna decomposers |
| Oribatida | *Steganacarus herculeanus* | Willmann, 1953 | Phthiracaridae | Mesofauna decomposers |
| Oribatida | *Steganacarus magnus* | (Nicolet, 1855) | Phthiracaridae | Mesofauna decomposers |
| Oribatida | *Suctobelba altvateri* | Moritz, 1970 | Suctobelbidae | Mesofauna predators |
| Oribatida | *Suctobelba trigona* | (Michael, 1888) | Suctobelbidae | Mesofauna predators |
| Oribatida | *Suctobelbella* sp. |  | Suctobelbidae | Mesofauna predators |
| Oribatida | *Tectocepheus minor* | Berlese, 1903 | Tectocepheidae | Mesofauna decomposers |
| Oribatida | *Tectocepheus velatus* subsp. *alatus* | Berlese , 1913 | Tectocepheidae | Mesofauna decomposers |
| Oribatida | *Tectocepheus velatus* subsp. *sarekensis* | Trägårdh, 1910 | Tectocepheidae | Mesofauna decomposers |
| Oribatida | *Tectocepheus velatus subsp. velatus* | (Michael, 1880) | Tectocepheidae | Mesofauna decomposers |
| Oribatida | *Tritegeus bisulcatus* | (Grandjean, 1953) | Cepheidae | Mesofauna decomposers |
| Oribatida | *Xenillus tegeocranus* | (Hermann, 1804) | Xenillidae | Mesofauna decomposers |
| Prostigmata | *Trombidium* cf. *holosericeum* | (Linnaeus, 1758) | Trombidiidae | Macrofauna predators |
| Pseudoscorpiones | *Neobisium carcinoides* | (Hermann, 1804) | Neobisiidae | Macrofauna predators |
| Symphyla | *Scolopendrellopsis subnuda* | Hansen, 1903 | Scolopendrellidae | Macrofauna decomposers |
| Symphyla | *Scutigerella immaculatus* | (Newport, 1845) | Scutigerellidae | Macrofauna decomposers |
